# *Wolbachia* strains for disease control: ecological and evolutionary considerations

**DOI:** 10.1111/eva.12286

**Published:** 2015-07-20

**Authors:** Ary A Hoffmann, Perran A Ross, Gordana Rašić

**Affiliations:** Pest and Environmental Adaptation Research Group, School of BioSciences, Bio21 Institute, The University of MelbourneParkville, Vic., Australia

**Keywords:** *Aedes*, deployment issues, disease control, fitness, strain attributes, virus, *Wolbachia*

## Abstract

*Wolbachia* are endosymbionts found in many insects with the potential to suppress vectorborne diseases, particularly through interfering with pathogen transmission. *Wolbachia* strains are highly variable in their effects on hosts, raising the issue of which attributes should be selected to ensure that the best strains are developed for disease control. This depends on their ability to suppress viral transmission, invade host populations, persist without loss of viral suppression and not interfere with other control strategies. The potential to achieve these objectives is likely to involve evolutionary constraints; viral suppression may be limited by the ability of infections to spread due to deleterious host fitness effects. However, there are exceptions to these patterns in both natural infections and in novel associations generated following interspecific transfer, suggesting that pathogen blockage, deleterious fitness effects and changes to reproductive biology might be at least partly decoupled to achieve ideal infection attributes. The stability of introduced *Wolbachia* and its effects on viral transmission remain unclear, but rapid evolutionary changes seem unlikely. Although deliberate transfers of *Wolbachia* across species remain particularly challenging, the availability of strains with desirable attributes should be expanded, taking advantage of the diversity available across thousands of strains in natural populations.

## Introduction

There is currently a high level of interest in using *Wolbachia* to indirectly suppress the incidence of vectorborne human diseases such as malaria, dengue fever or filariasis (McGraw and O'Neill 2013; Sinkins 2013; Bourtzis et al. 2014), or plant diseases caused by mosaic viruses and other disease agents (Box 1). *Wolbachia* are endosymbiotic bacteria living in all orders of insects and in other invertebrates. They are often promoted as a ‘natural’ way of manipulating insect hosts, in contrast to other approaches for manipulating vector biology, particularly through genetic modification, that are often regarded with suspicion because they involve novel constructs that are not present in the environment with the potential to spread to other nonvector species. While *Wolbachia* are already widespread in the environment, they are restricted to living exclusively within host cells and spread by changing the biology of their host species (O'Neill et al. 1997).

**Box 1: List of species in which *Wolbachia* have been considered from an applied perspective**

**Table:** 

Human disease vectors	
Mosquitoes	Target pathogens
*Aedes aegypti*	*Viruses*: dengue, yellow fever, chikungunia, zika, West Nile *Nematodes*: filarial, mermithid
*Aedes albopictus*	*Viruses*: dengue, chikungunia, Eastern equine encephalitis, La Crosse, West Nile, Japanese encephalitis *Nematodes*: filarial
*Aedes polynesiensis*	*Viruses*: zika, dengue, Ross River *Nematodes*: filarial
*Culex pipiens* species complex	*Viruses*: West Nile, Eastern equine encephalitis, Venezuelan equine encephalitis, Japanese encephalitis, St. Louis encephalitis, Ross River, Murray Valley encephalitis, Rift valley *Nematodes*: filarial
*Anopheles stephensi*	Malaria parasites

Plant disease vectors	
Planthoppers	Target pathogens
*Laodelphax striatellus*	*Viruses*: barley yellow striate mosaic, Northern cereal mosaic, maize rough dwarf, rice stripe tenuivirus, rice black-streaked dwarf, wheat chlorotic streak
*Nilaparvata lugens*	*Viruses*: rice ragged, grassy stunt

Pests	
Moths	Hosts
*Ephestia kuehniella*	Grains, flour
*Ostrinia scapulalis*	Beans
*Cadra cautella*	Grains, dried fruits

Fruit flies	Hosts
*Ceratitis capitata*	Fruits
*Rhagoletis cerasi*	Fruits
*Bactrocera oleae*	Olive fruits

*Wolbachia* can be used in a variety of ways for disease suppression, by decreasing the size of a vector population through (i) the release of *Wolbachia*-infected males that are incompatible with females (O'Connor et al. 2012) or (ii) the invasion of a *Wolbachia* strain that produces deleterious fitness effects particularly under seasonally variable environments (Rašić et al. 2014a), and particularly by (iii) decreasing the ability of the vector population to transmit diseases through the invasion of a *Wolbachia* strain that interferes directly with transmission (Teixeira et al. 2008; Kambris et al. 2009; Moreira et al. 2009; Walker et al. 2011). The third option is considered particularly promising because it may not require ongoing management by health authorities; once a *Wolbachia* strain blocking disease transmission has invaded a target vector population by altering host reproduction, the *Wolbachia* strain should stay at a high frequency in that population without further releases being required (Hoffmann et al. 2011). It is also important to note that the three strategies are not mutually exclusive but rather complementary.

Despite the promise held by *Wolbachia*-based disease suppression programmes particularly for viral diseases spread by mosquito vectors (Box 1), there are still substantial challenges in their widespread deployment. In particular, strains of *Wolbachia* for release need to be carefully selected and evaluated to ensure long-term viability of the strategy in the face of ongoing evolutionary changes, and to meet any regulatory and community concerns. In this study, we focus on these issues, using information that has been collected on insects naturally infected with *Wolbachia* and on artificial introductions of *Wolbachia* into new hosts. We consider the development of strains and host backgrounds that combine desirable attributes for disease suppression with those required for rapid invasion into target vector populations, as well as the likely long-term evolutionary stability of effects generated by *Wolbachia* in these populations. Much of the information we discuss comes from research in *Drosophila* species where *Wolbachia* infections have been investigated within an evolutionary and ecological context since the early 80s, and where a large number of infections have been transferred across species to investigate the interaction and evolution of host and *Wolbachia* genomes.

## Diversity of *Wolbachia* and their effects

There is an enormous diversity of *Wolbachia* strains in nature. DNA sequence data have been used to demonstrate the presence of multiple *Wolbachia* variants within the same individual host, the presence of variation among *Wolbachia* sequences collected from different conspecific individuals, and molecular changes in the same *Wolbachia* infection when it is transferred to different host species. Most molecular comparisons have focussed on describing variation in *Wolbachia* infections across related species to characterize patterns of horizontal and vertical transmission of infections across time (e.g. Bing et al. 2014; Morrow et al. 2014), using sets of conserved primers for a series of genes such as the MLST set (Baldo et al. 2006). Many studies have used primer sets to demonstrate variation in *Wolbachia* strains within the same host. An example of a species carrying a complex of infections is the tsetse fly, where 37 different *Wolbachia* variants have been identified (Symula et al. 2013). Numerous *Wolbachia* strains have also been identified in mosquitoes of the *Culex pipiens* species complex (Atyame et al. 2011; Morningstar et al. 2012) where they (rather than the nuclear background) largely control patterns of cytoplasmic incompatibility (Duron et al. 2006). Variability among *Wolbachia* within the same host could arise through recombination and mutation (Klasson et al. 2009; Atyame et al. 2011), and a new *Wolbachia* strain could spread if it has a selective advantage and/or generates patterns of cytoplasmic incompatibility or other changes to host reproduction that favour its spread. Horizontal transmission of *Wolbachia* across hosts such as mediated through a parasitoid could also result in the introduction of a new *Wolbachia* strain into a host population. Once new *Wolbachia* strains arise, they can displace existing strains at a rapid rate, as indicated by the replacement of *w*Au by *w*Ri in Australian populations of *Drosophila simulans* (Kriesner et al. 2013), but these types of replacements are thought to occur very rarely (Richardson et al. 2012).

The majority of *Wolbachia* stains have undescribed effects, having been detected in organisms *via* molecular tools (Hilgenboecker et al. 2008; Ahmed et al. 2013) and not further studied experimentally. Many of these strains may well have little impact on their host, but nevertheless persist because of a high fidelity of vertical transmission from mothers to offspring. Such infections with no apparent phenotypic effects on hosts have been described in *Drosophila* species (e.g. Hoffmann et al. 1996). Other *Wolbachia* strains are likely to have dramatic effects on their host; the most widespread of these effects is cytoplasmic incompatibility, where *Wolbachia* presence leads to the death of embryos and sometimes immature offspring when infected fathers mate with uninfected mothers (or mothers carrying a different *Wolbachia* strain). There are also *Wolbachia* infections that cause the death of male offspring only (male killers) and others that lead to parthenogenetic reproduction in haplodiploid organisms or feminization of male offspring (reviewed in O'Neill et al. 1997). Even when *Wolbachia* appear to have no phenotypic effects on their host's reproduction, they might nevertheless have other effects that only become evident once appropriate host challenges are provided; for instance, the viral blocking activity of *Wolbachia* strains only became apparent once infected *Drosophila* strains were challenged with RNA viruses (Teixeira et al. 2008; Hedges et al. 2008, Osborne et al. 2009).

*Wolbachia* effects exerted on hosts typically fall along a continuum; for instance, cytoplasmic incompatibility can range from complete (all offspring die) as in the case of many *Wolbachia* infections from mosquitoes (e.g. Rasgon and Scott 2003), to relatively weak (a small proportion of offspring die) as in the case of particular *Drosophila* infections (e.g. Reynolds et al. 2003). Moreover, the effects of *Wolbachia* on hosts can change markedly depending on environmental conditions and the age of the insect. Factors, such as the presence of natural antibiotics (Clancy and Hoffmann 1998; Lu et al. 2012), temperature extremes (Mouton et al. 2007; Bordenstein and Bordenstein 2011), the age of the male and female (Unckless et al. 2009; Tortosa et al. 2010) and interactions among these factors (Mouton et al. 2007; Bordenstein and Bordenstein 2011), can all influence the density of *Wolbachia* in host tissues and host effects such as cytoplasmic incompatibility.

*Wolbachia* density often varies substantially among individuals under field conditions (e.g. Ahantarig et al. 2008). This variation could potentially influence the transmission, fitness effects and expression of cytoplasmic incompatibility, which has been characterized in detail in *Drosophila* populations where variability in cytoplasmic incompatibility is high (e.g. Turelli and Hoffmann 1995) and in *Culex* populations where the variability is low (Rasgon and Scott 2003). However, it is not clear whether the variability reflects *Wolbachia*/host genomic variation or environmentally induced variation that might only have a temporary effect on density and host phenotypes. For instance, when the *w*Ha infection in *D*. *simulans* was tested in multiple host lines derived from the field, variation in the ovarian density of the *Wolbachia* infection among host lines was maintained for several generations, but was eventually lost (Correa and Ballard 2012). Therefore, while experimental studies might indicate a clear correlation between *Wolbachia* density and cytoplasmic incompatibility/deleterious effects (e.g. Clancy and Hoffmann 1998), it is not clear whether density variation is necessarily linked to variation in the *Wolbachia* genome. Recently, a group of *Wolbachia* genes associated with density variation (the Octomom region) has been identified in the *w*MelPop strain of *D*. *melanogaster* (Chrostek and Teixeira 2015) and might provide candidates for affecting density in field samples.

### Unpredictable phenotypic effects in new hosts

A substantial number of *Wolbachia* strains have now been transferred through microinjection across species boundaries, particularly in the genus *Drosophila*, but also across genera within and among insect orders (Table 1, Appendix S1). Successful *Wolbachia* transfers can be challenging, although those involving *Drosophila* species have been undertaken for some time and have become fairly routine (e.g. Poinsot et al. 1998; Charlat et al. 2002). Cross-infection experiments where *Wolbachia* are artificially transferred from one species to another have demonstrated (particularly in *Drosophila*) that host effects associated with a particular *Wolbachia* strain can persist or be modified after transfer to a new host (e.g. Ikeda et al. 2003; Osborne et al. 2012; Veneti et al. 2012).

**Table 1:** Stable *Wolbachia* infections in native and transinfected hosts, their reproductive effects (CI – cytoplasmic incompatibility, MT – maternal transmission), fitness effects, and viral blocking effectiveness where demonstrated (? – information unavailable). Effect size is denoted as: high (>90%), moderate/partial (20–90%), low (<20%) and none (no detectible effects). More details are found in Appendix S1

Strain	NATIVE HOST	CI	MT	Fitness cost	Viral blockage	TRANSFECTED HOST	CI	MT	Fitness cost	Viral blockage
*w*Mel	*Drosophila melanogaster*^1^	Partial*,^1,2^ none^3^	Partial^4^	None^5,6^ some benefits^3,7^	High^8^ moderate^9,10^ low^8,9,10,11^ none^8^	*Aedes aegypti*^12^	High^12^	High^12^	Low^12,13^	High^12^ moderate^14,15^ low^14^
						*Ae. albopictus*^16^	High^16^	High^16^	None^17^	High^16,17^
						*D*. *simulans*^18^	High^18^	High^18^	?	Moderate^19,20^
*w*MelPop	*D*. *melanogaster*^21^	Partial*,^22^ none^21,23^	High^24^	High†,^21,22,24^	High†,^10,25^	*Ae. aegypti*^26^	High^26,27^	High^26,27^	High^26,27,28,29^	High^14,15,30^ moderate^30^
						*Ae. albopictus*^31^	Low^31^	Partial^31^	High^31^	?
						*D*. *simulans*^23^	High*,^23,24,32^	High^23,24,32^	High/attenuated^23,32^	?
*w*Au	*D*. *simulans*^33^	None^33^	High^33^	None^33^	High^19,20^	*D*. *melanogaster*^34^	Low/none^34^	High‡,^34^	Moderate^35^	High^35^
*w*MelCS	*D*. *melanogaster*^36^	Low*,^2^ none^37^	?	Moderate^10^ none^35,38^	Moderate^10,25^	*D*. *simulans*^20^	?	?	?	High^20^
*w*Inn	*D*. *innubila*^39^	*Male killing*^39^	High^39^	Some benefits^40^	Low^40^	*D*. *simulans*^41^	None^41^	High^41^	Some benefits^41^	None^41^
						*D*. *melanogaster*^41^	None^41^	Partial^41^	Some benefits^41^	?
*w*Ri	*D*. *simulans*^42^	Partial*,^42^	High^43^ partial^44^	Low^43^ attenuated^45^	Moderate^19^ low^19^	*Ae. albopictus*^46,47^	High^46^	High^46^	?	?
						*D*. *melanogaster*^48^	Low^48^	Partial^48^	?	?
						*D*. *serrata*^49^	High^49^	High^49^	?	?
						*D*. *yakuba complex*^50^	High^50^	High^50^	?	?
						*Laodelphax* *striatellus*^51^	High^51^	Partial^51^	?	?
*w*AlbB	*Ae. albopictus*^52^	High^53,54,55^	High^56^	None^57^ some benefits^54,55^	Low^17,58,59^	*Ae. aegypti*^60,61^	High^60^ partial^61^	High^60^ partial^61^	Moderate§ none^60^	High^62^
						*Ae. polynesiensis*^63^	High^63^	High^63^	Moderate^63^	Moderate^64^
*w*Pip	*Culex pipiens* *complex*^65,66^	High^67^	High^67^	Low^68^ none^67^	Low^8^	*Ae. albopictus*^69^	High^69^	High^69^	Low^69^ none^70^	?

^1^Hoffmann (1988); ^2^Reynolds and Hoffmann (2002); ^3^Fry et al. (2004); ^4^Hoffmann et al. (1998); ^5^Harcombe and Hoffmann (2004); ^6^Montenegro et al. (2006); ^7^Fry and Rand (2002); ^8^Glaser and Meola (2010); ^9^Teixeira et al. (2008); ^10^Chrostek et al. (2013); ^11^Rances et al. (2012); ^12^Walker et al. (2011); ^13^Hoffmann et al. (2014a); ^14^van den Hurk et al. (2012); ^15^Hussain et al. (2013); ^16^Blagrove et al. (2012); ^17^Blagrove et al. (2013); ^18^Poinsot et al. (1998); ^19^Osborne et al. (2009); ^20^Martinez et al. (2014); ^21^Min and Benzer (1997); ^22^Reynolds et al. (2003); ^23^McGraw et al. (2001); ^24^Carrington et al. (2009); ^25^Hedges et al. (2008); ^26^McMeniman et al. (2009); ^27^Yeap et al. (2011); ^28^McMeniman and O'Neill (2010); ^29^Turley et al. (2009); ^30^Moreira et al. (2009); ^31^Suh et al. (2009); ^32^Carrington et al. (2010); ^33^Hoffmann et al. (1996); ^34^Yamada et al. (2011); ^35^Chrostek et al. (2014); ^36^Solignac et al. (1994); ^37^Holden et al. (1993); ^38^Serga et al. (2014); ^39^Dyer and Jaenike (2004); ^40^Unckless and Jaenike (2011); ^41^Veneti et al. (2012); ^42^Hoffmann et al. (1986); ^43^Hoffmann et al. (1990); ^44^Turelli and Hoffmann (1995); ^45^Weeks et al. (2007); ^46^Xi et al. (2006); ^47^Fu et al. (2010); ^48^Boyle et al. (1993); ^49^Clancy and Hoffmann (1997); ^50^Zabalou et al. (2004); ^51^Kang et al. (2003); ^52^Sinkins et al. (1995); ^53^Dobson et al. (2001); ^54^Dobson et al. (2004); ^55^Dobson et al. (2002); ^56^Kittayapong et al. (2002); ^57^Calvitti et al. (2009); ^58^Mousson et al. (2012); ^59^Mousson et al. (2010); ^60^Xi et al. (2005); ^61^Ruang-Areerate and Kittayapong (2006); ^62^Bian et al. (2010); ^63^Andrews et al. (2012); ^64^Bian et al. (2013a); ^65^Hertig and Wolbach (1924); ^66^Yen and Barr (1973); ^67^Rasgon and Scott (2003); ^68^de Almeida et al. (2011); ^69^Calvitti et al. (2010); ^70^Moretti and Calvitti (2013).

* High for one day old males, but decreases rapidly with increasing male age;

† increases with higher Octomom copy numbers (Chrostek and Teixeira 2015);

‡ inferred based on routine propagation of transinfected lines without loss of infection over time;

§ unpublished work by J Axford and AA Hoffmann.

The marked changes in cytoplasmic incompatibility and other reproductive effects, as well as host fitness effects, are typified by the *w*Au infection and lack of fitness effects in its native host but life shortening following transfer to *D*. *melanogaster* (Chrostek et al. 2014), and the absence of male killing when *Wolbachia* from *Drosophila innubila* are transferred to *D*. *melanogaster* and *D*. *simulans* (Veneti et al. 2012). As another example, *w*CauA causes cytoplasmic incompatibility in its native host, *Cadra cautella* (Sasaki and Ishikawa 1999), but when transferred to *Ephestia kuehniella,* it causes male killing (Sasaki et al. 2002) (see Appendix S1). There are also several other instances where shifts in cytoplasmic incompatibility occur when *Wolbachia* from one host are transferred to a different species within the same genus (e.g. Boyle et al. 1993), and clearly, viral interference will also depend on host effects as reflected by the limited blockage provided by *w*AlbB in its native *Ae. albopictus* host compared to strong blockage when this infection is transferred to *Ae. aegypti* (Bian et al. 2010) and other examples (Table 1, Appendix S1).

## Desirable attributes of *Wolbachia* strains for disease suppression

With many thousands of *Wolbachia* strains existing in nature and interacting with host genomes and local environments in different ways, *Wolbachia* could be used in a variety of ways for disease control strategies aimed at suppressing vector populations and directly interfering with disease transmission. Some important transfers of *Wolbachia* to disease vectors have now been achieved, including transfers of *Wolbachia* from *Drosophila* to *Aedes* mosquitoes for the production of vectors that exhibit shortened lifespan (McMeniman et al. 2009) and suppression of RNA viruses and other disease agents (Kambris et al. 2009; Moreira et al. 2009; Walker et al. 2011). In addition, there have been successful transfers of *Wolbachia* from *Aedes albopictus* to *Aedes aegypti* to achieve virus suppression (Xi et al. 2005; Bian et al. 2010). These transfers capture a tiny fraction of the vast diversity of *Wolbachia* strains available in natural populations of insects related to mosquitoes. Yet, they are already raising questions about how *Wolbachia* strains and host backgrounds might be developed for disease suppression.

Different strain attributes are required by the three strategies that use *Wolbachia* to reduce disease transmission. The simplest requirement is for population suppression *via* male release where the main attribute is for released males to exhibit strong cytoplasmic incompatibility when they mate with field females. Released males also need to be competitive with males from natural populations. Competitive ability could be reduced if *Wolbachia* in the release strain directly reduces male competitive fitness and/or if the host nuclear background of the release strain has a detrimental effect on male field competitiveness. At least for *Ae.aegypti* carrying the *w*Mel or *w*MelPop infection, there is no evidence that *Wolbachia* directly reduces male competitive fitness (Segoli et al. 2014), while *Ae. polynesiensis* carrying *Wolbachia* are also competitive in field releases (O'Connor et al. 2012). Detrimental host nuclear effects might develop if the release strain evolves and becomes adapted to conditions used for artificial rearing. This can be circumvented through backcrossing the release strain to field-sourced material prior to releases taking place, although it may then be more difficult to rear the strain under the artificial conditions if adaptation has taken place. Male competitiveness also needs to be high for successful *Wolbachia* strategies involving invasion (that utilize deleterious fitness effects and viral interference) because strong cytoplasmic incompatibility is required to drive the infection into a target population. In addition, several other attributes will be required for invasion-based strategies.

### Ease of invasion into field populations

To produce disease suppression by interfering with pathogen transmission or expressing deleterious fitness effects, *Wolbachia* strains need to invade and reach high frequencies in focal populations. In *Wolbachia* strains that have so far been introduced into *Ae. aegypti* populations, cytoplasmic incompatibility has been complete or nearly complete with uninfected target populations (Xi et al. 2005; McMeniman et al. 2009; Walker et al. 2011; Yeap et al. 2011), facilitating invasions. As long as there are no substantial deleterious effects of the *Wolbachia* on the hosts and as long as the infection is transmitted with a relatively high fidelity, invasion should be possible under strong cytoplasmic incompatibility. However, if a focal population is already infected with a *Wolbachia* strain that shows bidirectional incompatibility with the release strain, invasion becomes more difficult. Under bidirectional incompatibility between two *Wolbachia* strains with equivalent deleterious effects on a host, the infection frequency of an introduced strain has to exceed 50% to achieve invasion (Hoffmann and Turelli 1997).This situation applies to the *w*Mel infection introduced into *Ae. albopictus* (Table 1) which is bidirectionally incompatible with the naturally occurring *Wolbachia* of this species (Blagrove et al. 2012). Invasion will also depend on other fitness attributes such as the ability of females carrying the *Wolbachia* strain to feed and locate breeding sites and the ability of larvae with the *Wolbachia* strain to compete against other conspecific larvae and other species.

### Reduced pathogen transmission

For effective suppression of vectorborne diseases (strategy (iii) from above), *Wolbachia* strains will need to directly interfere with pathogen transmission in vector species. In *Aedes* mosquitoes, this has often been assessed in laboratory-based assays where blood is mixed with virus cultures to mimic titres that might be found in infected humans (Moreira et al. 2009). However, it is ideally assessed by feeding mosquitoes directly on blood from infected humans and assessing pathogens in tissue through which transmission occurs, such as the salivary glands and saliva of mosquitoes (Ferguson et al. 2015).

The ability of *Wolbachia* to block viruses and other microbes will depend on the nature of the viruses and the *Wolbachia* strains. In *Drosophila*, it appears that some types of viruses (DNA viruses in particular) are not affected by the presence of *Wolbachia* in host cells, whereas RNA viruses appear to be inhibited (Teixeira et al. 2008). The extent of inhibition varies dramatically between *Wolbachia* strains, such that some strains cause a dramatic reduction of the viral load in the host, whereas others have little impact (Table 1). In *Aedes* mosquitoes where stable *Wolbachia* infections have been established, the potential for *Wolbachia* to block different dengue virus serotypes and other RNA viruses seems to be high (Table 1, Appendix S1). The *w*MelPop infection appears to be highly efficacious in blocking different dengue serotypes as well as other arboviruses, at least in laboratory-based assays (Moreira et al. 2009; van den Hurk et al. 2012). For other *Wolbachia* infections, particularly *w*Mel and *w*AlbB, blockage against dengue serotypes also appears robust (Bian et al. 2010; Frentiu et al. 2014), but somewhat weaker than provided by *w*MelPop (Walker et al. 2011). Recent data for *w*Mel feeding on blood from infected human patients also point to strong blockage of dengue in saliva but show some differences among serotypes (Ferguson et al. 2015).

### Stable effects on hosts

Once a high frequency of infection is reached through releases and subsequent invasion driven by cytoplasmic incompatibility and other effects, *Wolbachia* effects on hosts and on viral transmission need to be stable, even if there are evolutionary changes in the virus and/or changes in the host's nuclear genome and *Wolbachia* genome. Data on the stability of *Wolbachia* effects following deliberate introductions are only just starting to emerge (Frentiu et al. 2014; Hoffmann et al. 2014a), but there is some relevant information from natural *Wolbachia* infections in other systems and particularly in *Drosophila* (Chrostek et al. 2013). Strategies that utilize the deleterious host effects associated with *Wolbachia* infections (strategy ii from above) also require that such effects remain stable even when there might be strong selection in the host genome to counter them.

## Evolutionary changes in the host genome

Evolution of host genomes in response to *Wolbachia* is certainly possible and is dramatically illustrated by the changes that nullify male killing by a natural *Wolbachia* infection in the butterfly *Hypolimnas bolina* (Hornett et al. 2006). Other relevant sources of evidence for such changes include experimental populations and longitudinal studies of natural populations.

Phenotypic changes in the expression of *Wolbachia* effects due to changes in the host nuclear genome have been documented in experimental host populations maintained both with and without deliberate selection pressures. These include evidence for nuclear-based attenuation of *w*MelPop effects on longevity in *D*. *melanogaster* hosts (Carrington et al. 2009) and in the novel host *D*. *simulans* (Carrington et al. 2010). When the *w*MelPop infection was transferred from *D*. *melanogaster* to *D*. *simulans*, it initially caused large fitness effects such as reducing fecundity and decreasing longevity as in its native host (McGraw et al. 2002). However, these effects attenuated quickly (Reynolds et al. 2003), such that *w*MelPop-infected *D*. *simulans* eventually exhibited an increase in longevity in some genetic backgrounds (Carrington et al. 2010). In *Ae. aegypti* mosquitoes, host genome changes can cause a decrease in deleterious effects of the introduced *w*MelPop on egg viability (A. Callahan and A. A. Hoffmann, unpublished data). The impact of host nuclear genomic backgrounds on virus blocking by *Wolbachia* has not yet been systematically investigated within either *Drosophila* or mosquito species. However, because the upregulation of immune response genes seems to be restricted to recently transferred infections in mosquitoes rather than native infections, an eventual decrease in blockage might be expected, given the likely high cost of constitutive immune gene expression.

The deliberate release of *Wolbachia* infections into natural mosquito populations provides an opportunity to test for host nuclear responses in natural populations across a period of a few years. In particular, the release of *w*Mel into uninfected *Ae. aegypti* populations in 2011 in two areas around Cairns, Australia (Hoffmann et al. 2011), provided an opportunity to monitor changes in both the viral interference effect and deleterious host effect across a three-year time span. These comparisons have indicated that dengue interference was not altered within this period (Frentiu et al. 2014) and neither were fitness effects of *Wolbachia* on its host (Hoffmann et al. 2014a). Because there is ongoing gene flow into these populations as inferred from infection frequencies and a lack of maternal leakage (Hoffmann 2014b), changes in the nuclear genome due to *Wolbachia* are only expected if selection is relatively strong.

Although the host genome can have a substantial effect on the expression of cytoplasmic incompatibility, deleterious effects and viral interference, it is not yet clear whether there will be rapid changes in the host genome that might affect the success of *Wolbachia* releases aimed at disease suppression. The most rapid host changes are expected in response to any deleterious effects induced by *Wolbachia*, whereas selection for altered effects of *Wolbachia* on viral interference should be weak unless the virus has a particularly large impact on host fitness (in which case selection would favour ongoing interference by *Wolbachia*). The host genome is therefore most likely to influence the success of a suppression strategy based on the expression of deleterious effects following invasion.

## Evolutionary changes in the *Wolbachia* genome

Evidence for possible changes in the *Wolbachia* genome comes from analysis of changes in laboratory and natural populations. In addition, the phenotypic effects associated with particular *Wolbachia* strains that are maintained following interspecific transfers (as in the case of the *w*MelPop infection following transfer from *D*. *melanogaster* to *D*. *simulans* and *Ae. aegypti* – Table 1) also point to effects on hosts mediated by the *Wolbachia* genome rather than the host genome.

It is still difficult to predict whether genomic changes in *Wolbachia* will be rapid enough to be detectable in experimental populations. For the virulent *w*MelPop infection, there have only been minor genomic changes since its introduction from *D*. *melanogaster* into the new host *Ae. aegypti* (Woolfit et al. 2013). On the other hand, in laboratory *D*. *melanogaster* populations, Octomom copy number seems to be able to evolve rapidly to alter the density of *w*MelPop (Chrostek and Teixeira 2015). There is also evidence from comparisons of conspecific populations of *D*. *melanogaster* that interactions between *w*Mel *Wolbachia* and host genomes can evolve fairly rapidly (Olsen et al. 2001; Fry et al. 2004). The *w*Ri infection of *D*. *simulans* is another such example (Weeks et al. 2007). The deleterious effects of this infection on female reproduction were first characterized in the late 1980s (Hoffmann et al. 1990). Twenty years on, such effects were no longer evident, and some infected females even showed a fecundity advantage over uninfected hosts, largely attributable to changes in *w*Ri or another maternally inherited factor (Weeks et al. 2007).

These findings suggest that while there is ample evidence for variation in the *Wolbachia* genome resulting in multiple strains of *Wolbachia* occurring in the same host and/or conspecific individuals carrying different *Wolbachia* strains, it is not clear whether there will be rapid changes in *Wolbachia* strains being released for disease suppression. As in the case of host genome changes, any changes will most likely lead to *Wolbachia* strains that exert a reduced deleterious effect on their host, which might only indirectly influence viral interference.

## Evolutionary changes in the viral genome

While viruses evolve rapidly, changes in the virus genome in response to *Wolbachia* are largely unpredictable due to a lack of relevant background information and clarity around selective factors involved (Bull and Turelli 2013). Selection on viral resistance to the blocking effects of *Wolbachia* might be expected, particularly given that there are differences in the extent to which dengue serotypes are blocked by *Wolbachia* (Frentiu et al. 2014; Ferguson et al. 2015). However, only some types of interactions between *Wolbachia* and viruses (such as direct competition between viruses and *Wolbachia*) are expected to lead to evolutionary changes (Bull and Turelli 2013). Moreover, viral evolutionary dynamics are affected by a number of factors unconnected to *Wolbachia* that drive viral strain replacements (Vu et al. 2010; Lambrechts et al. 2012). *Wolbachia* and/or host genomes could also evolve in response to any changes in the virus, particularly if these affect the fitness of the vector host, although (at least in the case of dengue) viral effects on hosts remain unclear (Maciel-de-Freitas et al. 2011).

## Other effects of *Wolbachia*

Even though *Wolbachia* can decrease transmission of many viral infections, its effects on others remain uncertain. A comparison of *Wolbachia-*infected and cured *D*. *melanogaster* strains and *Culex quinquefasciatus* strains suggested that *Wolbachia* might block West Nile virus (Glaser and Meola 2010). However, most *Culex quinquefasciatus* populations appear naturally infected with *Wolbachia* but are still capable of transmitting West Nile (Micieli and Glaser 2014). This may reflect the fact that *Wolbachia* densities in natural infections are too low to have much impact on transmission of this virus. On the other hand, in a recent study where *Wolbachia* from another mosquito were injected into *Culex dorsalis* females, the titre of West Nile virus increased (Dodson et al. 2014), although this may have been an effect of the infection process; the effect of *Wolbachia* on West Nile needs to be investigated in a host mosquito species carrying a stably introduced *Wolbachia* infection. In *Spodoptera* moths, *Wolbachia* may also increase susceptibility to a virus (Graham et al. 2012); infection by nucleopolydrovirus was associated with moths carrying different strains of *Wolbachia*, and laboratory tests with one of the *Wolbachia* strains (likely a male killer) indicated much higher mortality levels following the viral infection. Because nucleopolydrovirus is being explored as a potential biopesticide, this result might point to a potentially novel application of *Wolbachia* releases for pest control.

It is not yet clear whether *Wolbachia*-based strategies will be effective against microbes other than viruses. *Wolbachia* introduced into the major malaria vector *Anopheles stephensi* protects against *Plasmodium* to some extent (Bian et al. 2013b), although perhaps insufficiently to provide much impact on disease transmission (Killeen et al. 2013). Moreover, it has been suggested that the presence of *Wolbachia* may even enhance the incidence of malaria pathogens to some extent (Zélé et al. 2014) although this requires further validation. In *Drosophila*, *Wolbachia* infections appear to have few consistent effects on bacterial infections (Wong et al. 2011), while in mosquitoes, it has been suggested that any effects on bacteria will depend on whether the immune system is upregulated following *Wolbachia* transfer (Ye et al. 2013).

Another issue relevant to disease transmission is the potential interaction between *Wolbachia* and pesticide susceptibility. For *Ae. aegypti* mosquitoes that are artificially infected with *Wolbachia*, the infection does not affect susceptibility to commonly used insecticides (Endersby and Hoffmann 2013). However, in *Culex pipiens* naturally infected with *Wolbachia*, there was rapid evolutionary increase of *Wolbachia* density in an insecticide-resistant line (Echaubard et al. 2010), suggesting a dynamic interaction between the *Wolbachia* and/or host genomes evolving under insecticide exposure.

Because most *Wolbachia-*transfected lines originate from few or just one female (Xi et al. 2005; McMeniman et al. 2009), *Wolbachia* invasions can cause a dramatic reduction of mitochondrial haplotype diversity within and among populations (H. L. Yeap and A. A. Hoffmann, unpublished data; Armbruster et al. 2003). There is a growing body of evidence linking the mitochondrial polymorphisms with differences in metabolic rate and some fitness components in *Drosophila* (e.g. Ballard et al. 2007; Kurbalija Novičić et al. 2015), suggesting that mitochondrial diversity in natural populations is maintained by natural selection. Mitochondrial variation might play an important role in the epistatic interaction between the mitochondrial and nuclear genomes in determining insect metabolic rate under varying environmental conditions (Arnqvist et al. 2010). It is therefore possible that the loss of mitochondrial diversity following *Wolbachia* invasion could affect the performance of infected populations.

Finally, the various *Wolbachia* effects on host fitness could change the size and age distribution of the mosquito larval community in containers (Mains et al. 2013). These effects in turn might influence interspecific interactions, particularly under high-density larval conditions when fitness differences between *Wolbachia*-infected strains and uninfected strains can become accentuated (Ross et al. 2014). These ecological effects of *Wolbachia* need to be evaluated following invasions into natural communities and could have a substantial effect on disease transmission if vector populations become suppressed due to the detrimental effects of *Wolbachia* infection. The most dramatic example involves the *w*MelPop infection of *Ae. aegypti*, which reduces the viability of eggs when held in a dried state (Yeap et al. 2011). During a dry season, this effect could result in the complete collapse of an isolated population until there is a reinvasion from another source (Rašić et al. 2014a). Population cage experiments indicate that collapse is likely in populations that are completely *Wolbachia*-infected (S. Ritchie unpublished data).

## A pathogen interference/spread trade-off?

**Figure 1:**
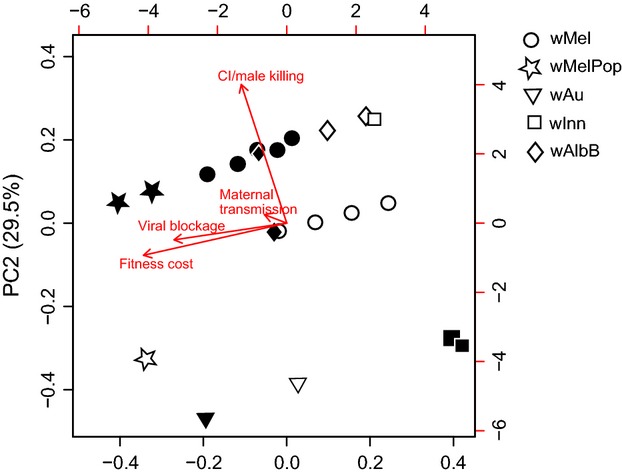
The first two principal components explaining 80.6% of total variation among *Wolbachia* strains in natural and transinfected hosts for the levels of: cytoplasmic incompatibility/male killing, maternal transmission, fitness costs and blockage of RNA viruses. Empty symbols denote natural *Wolbachia* infections, and filled symbols denote transinfections. Each infection attribute is ordered as: 0 (no effect), 1 (low), 2 (medium/partial) or 3 (high/full effect). Fitness cost has an additional value of −1 for infection effects that are somewhat beneficial. Twenty-one data points summarize values extracted from Table 1. Overall effects in natural hosts seem different from those in transinfected hosts, and the effects are also virus-dependent. Colinearity between fitness cost and viral blockage suggest that there is a possible trade-off between these effects, such that strains with strong viral protection might be difficult to spread due to higher deleterious effects on the host. Exceptions to these patterns, ho wever, indicate that it may be possible to achieve a desirable combination of infection attributes, but more strains need to be tested.

It is possible that *Wolbachia* infections that provide the strongest blockage of pathogen transmission might not spread easily into populations (Fig. 1). This possibility arises because a high density of *Wolbachia* in hosts may increase viral blockage but decrease host fitness (Chrostek et al. 2013; Sinkins 2013; Martinez et al. 2014), and such a trade-off could have driven past cycles of *Wolbachia* strain replacements in natural populations. For instance, the *w*Mel-CS strain in *D*. *melanogaster* which causes strong virus blockage (Table 1) may have been replaced with the *w*Mel strain which causes weaker blockage but does not decrease longevity to the same extent in this host (Chrostek et al. 2013). Relevant information to explore the notion of such a trade-off comes from (i) comparisons of viral suppression, host fitness and *Wolbachia* density between infected hosts, (ii) inferences from natural populations and (iii) mechanistic understanding of the common basis of viral interference.

### Viral suppression vs host fitness and *Wolbachia* density

Several authors have contrasted viral blockage (measured as survival/longevity following pathogen infection) in *Wolbachia* strains from *Drosophila* with effects on host fitness (mostly measured as longevity in the absence of the infection) and on cytoplasmic incompatibility (Table 1). These comparisons involve a relatively limited number of *Wolbachia* infections and a comparison of natural and introduced *Wolbachia* strains which may have different dynamics (Table 1, Fig. 1). In *D*. *simulans*, where the largest number of comparisons have been made involving 19 strains, survival following RNA viral infection is positively correlated with *Wolbachia* tissue density, although there are strains with relatively high *Wolbachia* densities that have a limited impact on survival (Martinez et al. 2014). Any association between deleterious *Wolbachia* effects and viral blockage may also not be particularly strong. In a comparison of five *Wolbachia* strains including one from a non-native host (*D*. *melanogaster*), the *w*Au infection caused the strongest blockage and had the highest density across tissues (Osborne et al. 2009, 2012), yet this strain does not cause detectable cytoplasmic incompatibility or have deleterious fitness effects, and is also transmitted at a high fidelity under field conditions (Hoffmann et al. 1996).

The *w*MelPop infection was transferred from *D*. *melanogaster* to *Ae. aegypti* to generate a strain that has a reduced longevity and thereby a reduced propensity to transmit diseases requiring a long incubation period through older females (McMeniman et al. 2009). In subsequent experiments, this strain was shown to have very strong blockage of viral replication and disease transmission in laboratory assays (Moreira et al. 2009). However, the *w*MelPop infection also causes substantial fitness costs, not just to longevity but also for egg viability, particularly when eggs are in a quiescent stage (McMeniman and O'Neill 2010; Yeap et al. 2011). The *w*MelPop infection also has deleterious effects on larval development under crowded conditions (Ross et al. 2014) and on some adult traits (e.g. Turley et al. 2009). In contrast, the *w*Mel infection causes somewhat weaker blockage of dengue and other viruses than *w*MelPop, but has fewer deleterious effects as well as having a lower titre in adults (Walker et al. 2011; Hoffmann et al. 2014a).

The *w*Mel infection was also transferred to *Ae. albopictus* where it causes strong blockage of chikungunya virus and dengue in laboratory assays, but has no apparent effects on longevity, hatch rates or other laboratory fitness parameters, despite generating strong cytoplasmic incompatibility (Blagrove et al. 2012, 2013). The *w*AlbB infection that blocks the transmission of dengue viruses in *Ae.aegypti* (Xi et al. 2005; Bian et al. 2010) has deleterious fitness effects on its host including a decrease in the viability of quiescent eggs and a reduction in longevity, although these deleterious effects are weaker compared to those exerted by *w*MelPop (J. Axford, unpublished data). When the native *w*PolA infection in *Ae. polynesiensis* was replaced with *w*AlbB from *Ae. albopictus*, there was an increase in *Wolbachia* density and evidence of dengue blocking in this species (Bian et al. 2013a), although it is not yet clear whether this transferred strain also produced deleterious fitness effects (Table 1).

*Wolbachia* density represents a complex phenotype, typically measured in three contexts: (i) whole body density, usually measured in newly eclosed adults; (ii) tissue specific density, focussing on heads, abdomens, ovaries, testes, salivary glands and so on; and (iii) age-specific (and life stage-specific) density, which can indicate whether *Wolbachia* continue to replicate when hosts have reached maturity or enter a quiescent phase. Changes in whole body density through exposure to low levels of antibiotics (usually tetracycline) typically reduce cytoplasmic incompatibility induced by *Wolbachia*, as demonstrated in the case of *D*. *simulans* (Clancy and Hoffmann 1998) and *Nasonia* wasps (Breeuwer and Werren 1993), and also reduce viral interference as shown for *w*Au in *D*. *simulans* (Osborne et al. 2012). These experimental data support the notion that differences in *Wolbachia* density can be linked to the expression of host effects and support the notion of a blocking/spread trade-off, particularly given that strain variation in *Wolbachia* density has a positive relationship to blockage in *D*. *simulans* as noted above (Martinez et al. 2014). However, the expression of strong cytoplasmic incompatibility in the *Drosophila paulistorum* species complex involves very low *Wolbachia* titres that can only be detected although nonconventional molecular methods (Miller et al. 2010), whereas high-density infections of other *Drosophila* species such as *w*Au (Osborne et al. 2012) have no detectable effects on cytoplasmic incompatibility or host fitness (Hoffmann et al. 1996). The effects of some infections can therefore be unconnected to their overall densities in hosts.

The tissue distribution of strains may influence pathogen blocking and host effects. For instance, the *w*Ri and *w*Ha infections in *D*. *simulans* are restricted mostly to gonadal tissues (Binnington and Hoffmann 1989; Correa and Ballard 2014), have mild deleterious effects (Hoffmann et al. 1990; Turelli and Hoffmann 1995) and cause mid- to low-level viral blockage (Osborne et al. 2009). On the other hand, the *w*Au and *w*MelPop infections may block pathogens effectively because they are found in a variety of tissues (Min and Benzer 1997; Osborne et al. 2012). In mosquitoes, *Wolbachia* presence in a variety of tissues through which a virus needs to pass to be transmitted may be crucial for generating strong transmission blockage; for instance, *w*MelPop which causes strong blockage is found in many tissues including the salivary glands of *Ae. aegypti* (Moreira et al. 2009). This feature seems particularly important for dengue viruses, where a density-dependent cellular relationship between *Wolbachia* and viral load has been reported (Lu et al. 2012).

Some *Wolbachia* infections attain higher densities at eclosion and replicate at a higher rate than others when hosts reach adulthood (Chrostek and Teixeira 2015), resulting in very high densities throughout the body as hosts age. While this high density might result in strong pathogen blockage, it could also eventually kill the host and limit the potential of such infections to spread. The reduced longevity of *D*. *melanogaster* infected by the *w*MelPop strain is thought to be due to ongoing replication and increasing density of this virus (Min and Benzer 1997), as is the reduction in longevity and increased mortality of quiescent eggs in *Ae. aegypti* artificially infected by *w*MelPop (McMeniman and O'Neill 2010; Yeap et al. 2011). Continued *Wolbachia* replication may also contribute to hybrid sterility in crosses between *D*. *paulistorum* semi-species (Miller et al. 2010).

The distribution of *Wolbachia* within hosts is expected to be altered due to evolutionary changes in the host and *Wolbachia*. The distribution of *Wolbachia* densities across tissues in long-standing infections is expected to become more variable if there is no evolution towards obligate relationship with the host (Correa and Ballard 2014). Strong cytoplasmic incompatibility with infected sperm should favour accurate transmission of an infection across generations, resulting in strong tissue tropism. However, for old infections where cytoplasmic incompatibility is weak (e.g. *w*Ma in *D*. *simulans*), *Wolbachia* density in tissues is expected to be variable because selection pressures for accurate transmission are weak (Correa and Ballard 2014). Such evolutionary changes are expected to weaken any blocking/spread trade-off.

These examples provide some support for a possible relationship between viral blockage, deleterious host effects and *Wolbachia* density, but too few strains have so far been examined. Moreover, the *Drosophila* data suggest that it is possible to identify infected lines demonstrating strong blockage, strong cytoplasmic incompatibility and no apparent fitness effects on the host. However, it is not yet clear whether such lines can be developed from novel combinations of hosts and infections generated through artificial transfers of *Wolbachia*.

### Inferences from changes in natural populations

Although the potential benefits that hosts gain from pathogen blocking have so far only been demonstrated in laboratory tests (Chrostek et al. 2013), it seems likely that similar benefits will occur under field conditions. Recently, the *w*Au infection in *D*. *simulans* which causes strong viral blockage but no detectable cytoplasmic incompatibility (Hoffmann et al. 1996) has nevertheless been shown to increase rapidly in natural populations (Kriesner et al. 2013), suggesting that the infection provides a fitness advantage to its host which may include viral blocking. Another example is the *w*Mel infection of *D*. *melanogaster*, which exhibits a stable cline in eastern Australia suggestive of selection (Hoffmann et al. 1994), but causes only partial cytoplasmic incompatibility in matings with young males (Reynolds et al. 2003). Given that this infection shows incomplete maternal transmission, it is hard to explain its persistence in *D*. *melanogaster* populations without assuming some sort of fitness benefit (Hoffmann et al. 1994). However, we still lack field data testing for a direct association between *Wolbachia* infection and natural viral load. If field strains exist that provide a fitness advantage under a high viral load but have few other effects on hosts, these would indicate that a blocking/spread trade-off can be avoided.

### Mechanistic understanding of viral interference/host effects – immune priming and other effects

If the mechanisms involved in viral blockage, cytoplasmic incompatibility, and host fitness effects were understood, it might help in predicting likely interactions among *Wolbachia* effects. Viral blocking by *Wolbachia* seems to involve a number of subcomponents (Rances et al. 2013; Sinkins 2013). Part of the blockage may come from the upregulation of the immune system, as suggested by the increased expression of some immune response genes following recent *Wolbachia* transfers in mosquitoes (Kambris et al. 2009; Lu et al. 2012). However, cross-species transfers of *Wolbachia* do not necessarily lead to immune priming, as in the case of the experimental *w*Au infection of *D*. *melanogaster* (Chrostek et al. 2014). Other mechanisms have also been implicated, such as competition for resources such as cholesterol, interactions involving various metabolites, and the expression of microRNAs (Caragata et al. 2013; Zhang et al. 2013). Blockage mechanisms may be partly related to changes in the tissue distribution and density of *Wolbachia* particularly following transfer to a new host. For instance, native *Wolbachia* infections of *Ae. albopictus* have a relatively low density; the natural *w*AlbB infection of *Ae. albopictus* seems to cause some suppression of dengue and chikungunya viruses in its native host (Mousson et al. 2012). However, following transfer from *Ae. albopictus* into *Ae. aegypti,* the same infection develops a much higher density and blocking effect (Lu et al. 2012).

Overall, these different lines of evidence point to a complicated pattern of interaction between pathogen blockage, deleterious fitness effects and cytoplasmic incompatibility. Host effects are not necessarily tightly linked mechanistically or through density, and a trade-off between blockage and spread might exist when host effects are predominantly related to density, but might in other cases be circumvented (Fig. 1). The *Drosophila* data indicate that strains such as *w*Au with strong blockage, no deleterious effects, high densities and no cytoplasmic incompatibility exist in populations alongside strains such as *w*Ha that cause strong cytoplasmic incompatibility, but no blockage or large deleterious effects. A range of infections with different combinations of attributes occur in natural populations, including strains that might exhibit relatively strong blockage while also being able to easily spread in the absence of over replication after eclosion, and a high density in reproductive tissues to ensure strong cytoplasmic incompatibility and high maternal transmission. Unfortunately, the same combination of attributes might not be maintained after such a strain is transferred to a target vector host. For example, the *w*Mel infection causes weak cytoplasmic incompatibility in its native *Drosophila* host but complete cytoplasmic incompatibility once transferred to *Ae. aegypti,* which has been essential for its successful spread (Hoffmann et al. 2011). Similarly, the *w*Au infection has no detectable fitness effect in its native host *D*. *simulans,* but causes a sharp reduction in lifespan and exhibits exponential growth when transferred to *D*. *melanogaster* (Chrostek et al. 2014). Therefore, intra- and intergeneric transfers across host species have unexpected consequences that may affect the suitability of strains for disease suppression.

## Other deployment issues

### Host population ecology

The successful invasion of *Wolbachia* infections will depend on the ecology of the host population. For example, if *w*MelPop is released into a host mosquito population where breeding sites lead to rapid egg hatch and where larvae develop under low densities, *Wolbachia* is more likely to invade. This is because the *w*MelPop infection does not strongly affect host viability and development time under low-density conditions and in the absence of dry conditions (McMeniman and O'Neill 2010; Yeap et al. 2011). On the other hand, there are development time and viability costs when *w*MelPop-infected mosquitoes are reared at a high density in competition with uninfected larvae (Ross et al. 2014). High-density conditions coupled with an extended period of dry season will raise costs and the threshold *Wolbachia* frequency required for a *w*MelPop invasion (Hancock et al. 2011; Yeap et al. 2014).

Areas of high mosquito density could be identified through factors such as housing characteristics, distribution of breeding containers and so on if this information is available from past surveys. Such information can be used to inform local invasion rates (Hoffmann et al. 2014b) and potential pockets where uninfected mosquitoes might persist and require additional treatment. Local knowledge of the ecology of mosquito populations should be used to inform release strategies; for instance, breeding containers that fill only occasionally after rain may need to be treated to remove sources of uninfected mosquitoes.

Release programmes also need to take into account expected movement patterns of mosquitoes and variation in host density across the region. Information on natural movement patterns from mark-release experiments or genetic analyses of local populations (e.g. Harrington et al. 2005; Olanratmanee et al. 2013) can provide a picture of likely movement patterns. By characterizing thousands of SNP markers, a much higher level of resolution of population structure can be obtained, and the movement of related individuals across a region can also be followed (Rašić et al. 2014b).

*Wolbachia* invasion into an isolated uninfected population of a target host only occurs if *Wolbachia* frequencies consistently exceed a particular frequency set by the size of the deleterious effects of *Wolbachia* on its host, levels of cytoplasmic incompatibility and to a lesser extent by the fidelity of maternal transmission (Hoffmann and Turelli 1997; Turelli 2010). If deleterious host effects associated with *Wolbachia* infections are too large, *Wolbachia* invasion into target host populations becomes difficult and high infection frequencies might not be sustained even if invasion succeeds. Invasion and persistence become increasingly unlikely if there is ongoing immigration of uninfected individuals into a relatively small release area (Barton and Turelli 2011) and if there are fitness effects of *Wolbachia* that decrease the size of the target population, making reinvasion by uninfected mosquitoes more likely (Rašić et al. 2014a).

A benefit of releasing infections with at least some deleterious fitness effects is that infections are expected to remain contained within an area rather than spreading rampantly (Barton and Turelli 2011; Hancock and Godfray 2012). This prediction is consistent with field experience from *w*Mel releases around Cairns, Australia, where *w*Mel did not spread outside areas where they were released even though *Wolbachia* were occasionally detected in other areas (Hoffmann et al. 2011, 2014b). Spread only occurs relatively slowly through a continuous residential area and is likely to be stopped by barriers to movement and high-density areas occupied by uninfected mosquitoes (Barton and Turelli 2011; Hancock and Godfray 2012; Hoffmann et al. 2014b). Spread is much easier to achieve when a large area with a high host density has been invaded and the surrounding area has a low density; an increase in host density outside the invaded zone can stop *Wolbachia* spread, particularly if the invasion point is high (Barton and Turelli 2011), as in the case of *w*MelPop (Yeap et al. 2011). Moreover, invasions might then fail to persist with a moderate influx of migrants into a population (Hancock et al. 2011).

Although the host fitness costs associated with *Wolbachia* infections could be used to suppress and even eradicate mosquito hosts in some isolated areas (Rašić et al. 2014a), they provide challenges for the infection spreading in large and continuously distributed mosquito populations. So far, attempts to spread the high cost *w*MelPop infection into relatively isolated natural populations in Vietnam and northern Australia have failed, despite high release rates and some success in getting the infection to a high frequency (T. H. Nguyen, unpublished data). The *w*MelPop infection did successfully invade semi-field population cages, but only when release rates were high and sustained for many weeks (Walker et al. 2011). Several strategies could assist in spreading infections with high deleterious effects, such as through the suppression of host populations across all life stages just prior to release (Hoffmann 2014), through the release of male-biased sex ratios (Hancock et al. 2011) or through the use of pesticide resistance genes and application of pesticides during the release process (Hoffmann and Turelli 2013). These strategies should assist in introducing such infections into relatively isolated populations, but the infection is unlikely to spread further outside these areas (Barton and Turelli 2011).

### Community acceptance

Although the likely benefits and costs of *Wolbachia*-based strategies for disease suppression can be identified to some extent, the final strategy and strain adopted will also depend on community acceptance and regulatory approval. A challenge for *Wolbachia* releases aimed at invasion and replacement is that there will be a period of time when mosquito numbers are increased above background levels to ensure that the *Wolbachia* infection exceeds an invasion threshold. As long as there are no fitness costs associated with the infection, *Wolbachia* is expected to spread from a very low starting frequency (close to 0%) depending on stochastic factors, with a slow rate of spread initially (Jansen et al. 2008). This type of spread has been observed in natural infections of *D*. *simulans* where resident populations number in the millions (Kriesner et al. 2013). However, with a threshold frequency of around 20–30%, the *w*Mel invasion into uninfected *Ae. aegypti* required releases across 10 weeks, at which time adult numbers increased by a factor of 1.5–2 (Hoffmann et al. 2011; Ritchie et al. 2013). The period of time and relative increase in mosquito numbers required will be greater if infections are costly, and/or if the release material has a relatively low fitness.

While a 1.5–2 fold increase in mosquito numbers might seem trivial, particularly when only one mosquito species is being targeted in release areas where several species are likely to co-occur, implementation of such a strategy can be challenging. In most countries where dengue is endemic and attributable to *Ae. aegypti* mosquitoes which breed around houses, communities are encouraged to decrease the availability of breeding sites, removing containers that might hold standing water, treating containers with chemicals, and perhaps fogging an entire area with pesticides. Such combined programmes can be effective in reducing mosquito densities (Erlanger et al. 2008), but often there is little impact on mosquito populations due to factors such as cryptic breeding sites that cannot be easily targeted (Heintze et al. 2007; Eisen et al. 2009). These strategies can also generate additional problems such as the evolution of pesticide resistance in hosts (Maciel-de-Freitas et al. 2014). Furthermore, there is often a poor correlation between measures of mosquito numbers and disease incidence (Bowman et al. 2014), making it difficult to justify such campaigns. Nevertheless, while education and engagement campaigns can help increase acceptance of *Wolbachia* releases (McNaughton and Huong 2014), communities may be reluctant to participate in *Wolbachia* release programmes and regulatory authorities may be reluctant to approve strategies where there is a deliberate increase in mosquito numbers over a period of time.

This issue becomes particularly important where the *Wolbachia* strains being introduced have high invasion thresholds and therefore require high release numbers across an extended period of time. For instance, *w*MelPop failed to invade isolated field populations despite releases across several months where frequencies exceeded 70% (T. H. Nguyen, unpublished data). Even when this infection invaded semi-field cages, it required more than 80 days before the infection reached fixation in one cage, despite a starting infection frequency of 65% (Walker et al. 2011). In contrast, infections such as *w*Mel seem to invade quite readily, at least based on experience in Australia.

One of the advantages of *Wolbachia* releases is that they are not necessarily incompatible with other control programmes. For instance, during the 2011 release of *w*Mel around Cairns, Australia, pesticides were applied by the health authorities to a residential block within the release site where a dengue case had been reported, and this did not inadvertently affect the local rate of increase of *Wolbachia* (Hoffmann et al. 2011). In this case, both the resident uninfected population of *Ae. aegypti* and the released mosquitoes did not contain appreciable levels of insecticide resistance. In contrast, in many communities where there has been widespread application of pyrethroids and other insecticides over some time, resistance levels in uninfected *Ae. aegypti* are expected to be high (Ranson et al. 2009). In such cases, insecticide application during the release could lead to a preferential removal of the infected released mosquitoes. However, it should be possible to minimize this issue by backcrossing infected release stock to the local genetic background of a target population with high insecticide resistance.

Finally, when there are community concerns about release numbers increasing above background levels, suppression of mosquitoes prior to starting releases could help to alleviate community concerns, as well as speeding up *Wolbachia* invasions by increasing the frequency of *Wolbachia*, and by producing vacant breeding sites for infected released females. In addition, it may be possible to release large numbers of nonbiting infected male mosquitoes to facilitate invasions when these males generate cytoplasmic incompatibility with uninfected mosquitoes (Hancock et al. 2011). Pesticide applications could also assist invasions if the release material carries a higher level of resistance than the resident population (Hoffmann and Turelli 2013). Although there is little risk that resistance alleles will spread to the uninfected resident populations as long as cytoplasmic incompatibility is complete and maternal transmission is high, this strategy is unlikely to be approved by regulators except in limited circumstances, for instance, where relevant genes are already present in a target population.

## Conclusions

Selecting a suitable strain of *Wolbachia* for release is not a straightforward process, and involves a balance between minimizing fitness costs while maximizing cytoplasmic incompatibility and blockage of disease agents, as well as considering community and regulatory issues. It is not yet clear to what extent desirable strain qualities can be combined or whether there are trade-offs that limit the options available. It seems essential to create and test a number of *Wolbachia* infections for releases, despite the challenges associated with this exercise that require thousands of microinjections to achieve success (McMeniman et al. 2009; Bian et al. 2013b). Nevertheless, there are many natural *Wolbachia* strains available within Diptera for potential introduction into disease vectors. Once a suitable strain has been identified, it will be necessary to monitor the long-term stability of the desirable effects because there may be further evolutionary changes in the host, *Wolbachia* and pathogen genomes that could modify *Wolbachia* effects, even though current data suggest they are relatively stable.

## Supporting Information

**Appendix S1.** Stable *Wolbachia* infections produced through microinjection, their effects on host reproduction and fitness, and potential blocking effectiveness where demonstrated.
