# Supplementary material for: Wolbachia strains for disease control: ecological and evolutionary considerations
Source: Evol Appl. 2015 Jul 20;8(8):751–68. doi: 10.1111/eva.12286 (PMC4561566; doi:10.1111/eva.12286)
Supplement: Supplementary file 1 — Appendix S1. Stable Wolbachia infections produced through microinjection, their effects on host reproduction and fitness, and potential blocking effectiveness where demonstrated. [file eva0008-0751-sd1.docx]

**Appendix 1.** Stable *Wolbachia* infections produced through microinjection, their effects on host reproduction and fitness, and potential blocking effectiveness where demonstrated. “?” denotes unavailable information. The level of pathogen blockage for each *Wolbachia* strain is defined as follows; High: the *Wolbachia* strain provides complete or nearly complete blockage of the pathogen. For example, the pathogen is absent or at low densities when *Wolbachia*-infected, or for pathogens that cause mortality, survival is unaffected by pathogen infection when also infected with *Wolbachia*. Low: statistically significant protection provided by *Wolbachia*, but pathogen densities remain high, or survival is only marginally improved when *Wolbachia*-infected. Moderate: intermediate protection that lies between the high and low categories. Yes: *Wolbachia* provides some level of protection, but the data are insufficient to determine the strength of protection. No: there is no significant difference between *Wolbachia-*infected and uninfected in terms of pathogen blockage. Reduced protection: *Wolbachia* infection decreases protection against the pathogen relative to uninfected. Abbreviations: CI, cytoplasmic incompatibility; MT, maternal transmission; DCV, Drosophila C Virus; FHV, Flock House Virus; DENV, Dengue Virus; WNV, West Nile Virus; YFV, Yellow Fever Virus; CHIKV, Chikungunya Virus; IIV-6, Insect Iridescent Virus 6; LACV, La Crosse Virus.

|  | **Native host** | | | | | **Transinfected Host** | | | | |
| --- | --- | --- | --- | --- | --- | --- | --- | --- | --- | --- |
| **Strain** | **Origin** | **Reproductive effect** | **Fitness effect** | **Pathogen** | **Protection** | **Transfer target** | **Reproductive effect** | **Fitness effect** | **Pathogen** | **Protection** |
| *w*Mel | *Drosophila melanogaster* [^1^](#_ENREF_1) | No CI [^2^](#_ENREF_2) or partial CI [^1^](#_ENREF_1)^,^ [^3^](#_ENREF_3)^,^ ^[[1]](#endnote-1)^  Incomplete MT [^4^](#_ENREF_4) | No clear effect [^5^](#_ENREF_5)^,^ [^6^](#_ENREF_6)  Enchanced fecundity [^2^](#_ENREF_2)^,^ [^7^](#_ENREF_7) and longevity [^2^](#_ENREF_2)^,^ [^8^](#_ENREF_8) | DCV ^[[2]](#endnote-2)^^,^ ^[[3]](#endnote-3)^  FHV ^b, c^  Nora virus ^[[4]](#endnote-4)^  IIV-6 ^b^  DENV ^b^  WNV ^b^  CHIKV ^b^  LACV ^b^  *Blauveria bassiana* ^c^  *Listeria monocytogenes,*  *Providencia rettgeri* ^b^  *Salmonella typhimurium* ^b, c^  *Erwinia carotorovra,*  *Burkholderia cepacia, Mycobacterium marinum* ^c^  *Leptopilina boulardi* ^[[5]](#endnote-5)^ | Moderate [^9^](#_ENREF_9)^,^ [^10^](#_ENREF_10)  Low [^9^](#_ENREF_9)^,^ [^10^](#_ENREF_10)  Yes [^9^](#_ENREF_9)  No [^9^](#_ENREF_9)  Low [^11^](#_ENREF_11)  High [^12^](#_ENREF_12)  Low [^12^](#_ENREF_12)  No [^12^](#_ENREF_12)  Yes [^13^](#_ENREF_13)  No [^14^](#_ENREF_14)  No [^14^](#_ENREF_14)^,^ [^15^](#_ENREF_15)  No [^15^](#_ENREF_15)  No [^16^](#_ENREF_16) | *Aedes aegypti* [^17^](#_ENREF_17) | Complete CI [^17^](#_ENREF_17)  Complete MT [^17^](#_ENREF_17) | Minimal cost [^17^](#_ENREF_17)  Fecundity and longevity costs [^18^](#_ENREF_18)^,^ ^[[6]](#endnote-6)^ | DENV ^b^  CHIKV ^[[7]](#endnote-7)^  YFV ^b, g^  WNV ^b, g^  *Erwinia carotorovra* ^b, c^  *Burkholderia cepacia* ^b, c^  *Salmonella typhimurium* ^b, c^    *Mycobacterium marinum* ^b, c^ | Moderate [^19^](#_ENREF_19) or high [^11^](#_ENREF_11)^,^ [^17^](#_ENREF_17)^,^ [^20^](#_ENREF_20)  Yes [^21^](#_ENREF_21)  Low [^21^](#_ENREF_21)  Moderate [^22^](#_ENREF_22)  Moderate [^15^](#_ENREF_15)  No [^15^](#_ENREF_15)^,^ ^[[8]](#endnote-8)^  Low [^15^](#_ENREF_15)  No [^15^](#_ENREF_15) |
|  |  |  |  |  |  | *Aedes albopictus* [^23^](#_ENREF_23) | Complete CI [^23^](#_ENREF_23)  Complete MT [^23^](#_ENREF_23) | No clear effect [^24^](#_ENREF_24) | DENV ^b, g^  CHIKV ^b, g^ | High [^23^](#_ENREF_23)  High [^24^](#_ENREF_24) |
|  |  |  |  |  |  | *Drosophila simulans* [^25^](#_ENREF_25) | >98% CI [^25^](#_ENREF_25)  Complete MT [^25^](#_ENREF_25) | ? | DCV [^26^](#_ENREF_26)^,^ [^27^](#_ENREF_27)^, b, c^  FHV ^b, c^   *Pseudomonas aeruginosa, Serratia marcescens, Erwinia carotovora* ^c^ | Moderate [^26^](#_ENREF_26)^,^ [^27^](#_ENREF_27)  Moderate [^27^](#_ENREF_27)  No [^28^](#_ENREF_28) |
|  |  |  |  |  |  | *Drosophila melanogaster* [^29^](#_ENREF_29) | ? | ? | ? | ? |
| *w*MelPop | *Drosophila melanogaster* [^30^](#_ENREF_30) | No CI [^30^](#_ENREF_30)^,^ [^31^](#_ENREF_31), or partial CI [^32^](#_ENREF_32)^,^ [^33^](#_ENREF_33)^, a^ | Life-shortening [^30^](#_ENREF_30)^,^ [^32^](#_ENREF_32)^,^ [^34^](#_ENREF_34)^,^ ^[[9]](#endnote-9),^ ^[[10]](#endnote-10)^  No fecundity effect [^32^](#_ENREF_32)^,^ [^35^](#_ENREF_35) | DCV [^10^](#_ENREF_10)^,^ [^36^](#_ENREF_36)^, b, c^  FHV ^b^  *Erwinia carotorovra,*  *Burkholderia cepacia, Salmonella typhimurium,*  *Mycobacterium marinum* ^c^  *Leptopilina boulardi* ^e^ | High [^10^](#_ENREF_10)^,^ [^36^](#_ENREF_36)^, j^  High [^10^](#_ENREF_10)  No [^15^](#_ENREF_15)  No [^16^](#_ENREF_16) | *Aedes aegypti* [^37^](#_ENREF_37)^, l^ | Complete CI [^37^](#_ENREF_37)^,^ [^38^](#_ENREF_38)  Complete MT [^37^](#_ENREF_37)^,^ [^38^](#_ENREF_38) | Life-shortening [^37^](#_ENREF_37)^,^ [^38^](#_ENREF_38)  Reduced egg viability in quiescent state [^38^](#_ENREF_38)^,^ [^39^](#_ENREF_39)  Reduced blood feeding success and fecundity [^39^](#_ENREF_39)^,^ [^40^](#_ENREF_40) | DENV ^b^  CHIKV ^b^  *Plasmodium gallinaceum* ^b^  *Brugia pahangi* ^b^  YFV ^b, g^  WNV ^b, g^  *Erwinia carotorovra* ^b, c^    *Burkholderia cepacia* ^b, c^  *Salmonella typhimurium* ^b, c^    *Mycobacterium marinum* ^b, c^ | High [^17^](#_ENREF_17)^,^ [^19^](#_ENREF_19)^,^ [^41^](#_ENREF_41)  Moderate [^41^](#_ENREF_41)  High [^41^](#_ENREF_41)  Moderate [^42^](#_ENREF_42)  High [^21^](#_ENREF_21)  High [^22^](#_ENREF_22)  Moderate [^15^](#_ENREF_15)^,^ [^42^](#_ENREF_42)  Low [^15^](#_ENREF_15)  Moderate [^15^](#_ENREF_15)  Moderate [^15^](#_ENREF_15) |
|  |  |  |  |  |  | *Aedes albopictus* [^43^](#_ENREF_43) | Low or absent CI [^43^](#_ENREF_43)  Incomplete MT [^43^](#_ENREF_43)^,^ ^[[11]](#endnote-11)^ | Greatly reduced hatch rate [^43^](#_ENREF_43) | ? | ? |
|  |  |  |  |  |  | *Drosophila simulans* [^35^](#_ENREF_35) | >90% CI [^31^](#_ENREF_31)^,^ [^35^](#_ENREF_35)^,^ [^44^](#_ENREF_44)^, a^  Complete MT [^31^](#_ENREF_31)^,^ [^35^](#_ENREF_35)^,^ [^44^](#_ENREF_44) | Longevity ^i^, fecundity and egg viability costs that have attenuated over time [^34^](#_ENREF_34)^,^ [^35^](#_ENREF_35)^,^ [^44^](#_ENREF_44) | ? | ? |
|  |  |  |  |  |  | *Drosophila melan**ogaster* [^33^](#_ENREF_33)^,^ ^[[12]](#endnote-12)^ | Partial CI [^33^](#_ENREF_33)^, m^  Complete MT [^33^](#_ENREF_33) | Life-shortening [^33^](#_ENREF_33)^,^ ^[[13]](#endnote-13)^ | ? | ? |
| *w*MelCS | *Drosophila melanogaster* [^45^](#_ENREF_45) | No CI [^45^](#_ENREF_45)^,^ [^46^](#_ENREF_46) or partial CI [^3^](#_ENREF_3)^,^ [^47^](#_ENREF_47)^, a^  >90% MT [^7^](#_ENREF_7) | Life-shortening [^10^](#_ENREF_10) or no longevity effect [^48^](#_ENREF_48)  Minor fecundity cost [^7^](#_ENREF_7) | DCV ^b, c^  FHV ^b, c^    CrPV ^c^  *Pseudomonas aeruginosa* ^b, c^  *Serratia marcescens, Erwinia carotovora* ^c^ | Moderate [^10^](#_ENREF_10)^,^ [^36^](#_ENREF_36)^,^ [^48^](#_ENREF_48)  Moderate [^10^](#_ENREF_10)^,^ [^36^](#_ENREF_36)^,^ [^48^](#_ENREF_48)  High [^36^](#_ENREF_36)  No [^28^](#_ENREF_28)  No [^28^](#_ENREF_28) | *Drosophila simulans* [^27^](#_ENREF_27) | ? | ? | DCV ^b, c^  FHV ^b, c^ | High [^27^](#_ENREF_27)  High [^27^](#_ENREF_27) |
| *w*Ri | *Drosophila simulans* [^49^](#_ENREF_49) | Partial CI [^49^](#_ENREF_49)^,^ [^50^](#_ENREF_50)^,^ [^51^](#_ENREF_51)^, a^  Complete MT in the laboratory [^50^](#_ENREF_50)  Incomplete MT in the field [^52^](#_ENREF_52) | Rapid evolution from a minor fecundity cost [^35^](#_ENREF_35)^,^ [^50^](#_ENREF_50) to a minor benefit [^51^](#_ENREF_51)  Reduced size [^53^](#_ENREF_53) | DCV ^c^  FHV ^c^  *Pseudomonas aeruginosa, Serratia marcescens, Erwinia carotovora* ^c^  *Beauveria bassiana* ^c^  *Leptopilina heterotoma* ^e^  *Leptopilina boulardi* ^e^ | Moderate [^26^](#_ENREF_26)  Low [^26^](#_ENREF_26)  No [^28^](#_ENREF_28)    No [^53^](#_ENREF_53)  Reduced protection [^53^](#_ENREF_53)  No [^16^](#_ENREF_16) | *Aedes albopictus* [^54^](#_ENREF_54) and *Aedes albopictus* ^[55](#_ENREF_55),^ ^[[14]](#endnote-14)^ | >95% CI [^56^](#_ENREF_56)  ~90% MT [^56^](#_ENREF_56)  >80% Unidirectional CI [^55^](#_ENREF_55)^, n^ | ? | ? | ? |
|  |  |  |  |  |  | *Drosophila melanogaster* [^57^](#_ENREF_57) | Low CI [^57^](#_ENREF_57)  ~60% MT [^57^](#_ENREF_57) | ? | ? | ? |
|  |  |  |  |  |  | *Drosophila mauritiana* [^58^](#_ENREF_58) | >97% CI [^58^](#_ENREF_58) | ? | ? | ? |
|  |  |  |  |  |  | *Drosophila serrata* [^59^](#_ENREF_59) | >90% CI [^59^](#_ENREF_59)  ~90% MT [^59^](#_ENREF_59) | ? | ? | ? |
|  |  |  |  |  |  | *Drosophila simulans* [^58^](#_ENREF_58)^,^ [^60^](#_ENREF_60) | >90% CI [^58^](#_ENREF_58) | ? | ? | ? |
|  |  |  |  |  |  | *Drosophila yakuba* species complex [^61^](#_ENREF_61) | >80-95% CI [^61^](#_ENREF_61)  Complete MT [^61^](#_ENREF_61) | ? | ? | ? |
|  |  |  |  |  |  | *Laodelphax striatellus* [^62^](#_ENREF_62)^,^ ^[[15]](#endnote-15)^ | >90% CI [^62^](#_ENREF_62)  Incomplete MT [^62^](#_ENREF_62) | ? | ? | ? |
| *w*Au | *Drosophila simulans* [^63^](#_ENREF_63) | No CI [^63^](#_ENREF_63)  Complete MT [^63^](#_ENREF_63) | No clear effect [^63^](#_ENREF_63) | DCV ^b, c^  FHV ^b, c^  *Pseudomonas aeruginosa, Serratia marcescens, Erwinia carotovora* ^c^  *Leptopilina boulardi* ^e^ | High [^26^](#_ENREF_26)^,^ [^27^](#_ENREF_27)^,^ [^64^](#_ENREF_64)  High [^26^](#_ENREF_26)^,^ [^27^](#_ENREF_27)  No [^28^](#_ENREF_28)    Yes [^16^](#_ENREF_16) | *Drosophila melanogaster* [^65^](#_ENREF_65) | Low or absent CI [^65^](#_ENREF_65)^, i^ | Life-shortening [^48^](#_ENREF_48) | DCV ^b, c^  FHV ^b, c^ | High [^48^](#_ENREF_48)  High [^48^](#_ENREF_48) |
| *w*Ha | *Drosophila simulans* [^66^](#_ENREF_66) | >85% CI [^47^](#_ENREF_47)^,^ [^66^](#_ENREF_66)^,^ [^67^](#_ENREF_67)  Incomplete MT [^67^](#_ENREF_67) | No effect [^68^](#_ENREF_68) | DCV ^b, c^  FHV ^b, c^  *Pseudomonas aeruginosa, Serratia marcescens, Erwinia carotovora* ^c^ | No [^26^](#_ENREF_26)^,^ [^27^](#_ENREF_27)  No [^26^](#_ENREF_26)^,^ [^27^](#_ENREF_27)  No [^28^](#_ENREF_28) | *Drosophila simulans* [^60^](#_ENREF_60)^,^ [^69^](#_ENREF_69)^,^ [^70^](#_ENREF_70) | Partial CI [^60^](#_ENREF_60)^,^ [^70^](#_ENREF_70)  Incomplete MT [^60^](#_ENREF_60)^,^ [^70^](#_ENREF_70) | ? | ? | ? |
| *w*No | *Drosophila simulans* [^67^](#_ENREF_67)^,^ [^71^](#_ENREF_71) | >75% CI [^47^](#_ENREF_47)^,^ [^67^](#_ENREF_67)  Incomplete MT [^67^](#_ENREF_67) | No effect [^68^](#_ENREF_68) | DCV ^b, c^  FHV ^c^  *Pseudomonas aeruginosa, Serratia marcescens, Erwinia carotovora* ^c^ | No [^26^](#_ENREF_26)  No [^26^](#_ENREF_26)  No [^28^](#_ENREF_28) | *Drosophila simulans* [^70^](#_ENREF_70) | Partial CI [^70^](#_ENREF_70)  Incomplete MT [^70^](#_ENREF_70) | ? | ? | ? |
| *w*Yak | *Drosophila yakuba* [^61^](#_ENREF_61) | No CI [^61^](#_ENREF_61) | ? | ? | ? | *Drosophila simulans* [^72^](#_ENREF_72) | 21-26.5% CI [^72^](#_ENREF_72) | ? | DCV ^b, c^  FHV ^b, c^ | No [^27^](#_ENREF_27)  Low [^27^](#_ENREF_27) |
| *w*Tei | *Drosophila teissieri* [^61^](#_ENREF_61) | No CI [^61^](#_ENREF_61) | ? | ? | ? | *Drosophila simulans* [^72^](#_ENREF_72) | >90% CI [^72^](#_ENREF_72) | ? | DCV ^b, c^  FHV ^b, c^ | Low [^27^](#_ENREF_27)  Low [^27^](#_ENREF_27) |
| *w*San | *Drosophila santomea* [^61^](#_ENREF_61) | No CI [^61^](#_ENREF_61) | ? | ? | ? | *Drosophila simulans* [^72^](#_ENREF_72) | 21-26.5% CI [^72^](#_ENREF_72) | ? | DCV ^b, c^  FHV ^b, c^ | No [^27^](#_ENREF_27)  No [^27^](#_ENREF_27) |
| *w*Inn | *Drosophila innubila* [^73^](#_ENREF_73) | >95% MT [^73^](#_ENREF_73)  Male-killing [^73^](#_ENREF_73) | Increased fecundity [^74^](#_ENREF_74) | FHV ^c^ | Low [^74^](#_ENREF_74) | *Drosophila melanogaster* [^75^](#_ENREF_75) | No reproductive manipulation [^75^](#_ENREF_75)  Incomplete MT [^75^](#_ENREF_75) | No, or slight beneficial effect [^75^](#_ENREF_75) | ? | ? |
|  |  |  |  |  |  | *Drosophila simulans* [^75^](#_ENREF_75) | No reproductive manipulation [^75^](#_ENREF_75)  Complete MT [^75^](#_ENREF_75) | No, or slight beneficial effect [^75^](#_ENREF_75) | DCV ^b, c^  FHV ^b, c^ | No [^27^](#_ENREF_27)  No [^27^](#_ENREF_27) |
| *w*Mau | *Drosophila mauritiana* [^58^](#_ENREF_58) | No CI [^58^](#_ENREF_58) | Greatly increased fecundity [^76^](#_ENREF_76) | ? | ? | *Drosophila simulans* [^58^](#_ENREF_58) | No CI [^58^](#_ENREF_58) | ? | ? | ? |
| *w*Ana | *Drosophila ananassae* [^77^](#_ENREF_77) | <25% CI [^77^](#_ENREF_77) | No fecundity effect [^77^](#_ENREF_77) | ? | ? | *Drosophila simulans* [^27^](#_ENREF_27) | ? | ? | DCV ^b, c^  FHV ^b, c^ | Moderate [^27^](#_ENREF_27)  No [^27^](#_ENREF_27) |
| *w*Ara | *Drosophila arawakana* [^78^](#_ENREF_78) | ? | ? | ? | ? | *Drosophila simulans* [^27^](#_ENREF_27) | ? | ? | DCV ^b, c^  FHV ^b, c^ | Moderate [^27^](#_ENREF_27) Moderate [^27^](#_ENREF_27) |
| *w*Bai | *Drosophila baimaii* [^78^](#_ENREF_78) | ? | ? | ? | ? | *Drosophila simulans* [^27^](#_ENREF_27) | ? | ? | DCV ^b, c^  FHV ^b, c^ | No [^27^](#_ENREF_27)  No [^27^](#_ENREF_27) |
| *w*Bic | *Drosophila bicornuta* [^78^](#_ENREF_78) | ? | ? | ? | ? | *Drosophila simulans* [^27^](#_ENREF_27) | ? | ? | DCV ^b, c^  FHV ^b, c^ | No [^27^](#_ENREF_27)  No [^27^](#_ENREF_27) |
| *w*Bor | *Drosophila borealis* [^79^](#_ENREF_79) | Male-killing [^79^](#_ENREF_79) | ? | ? | ? | *Drosophila simulans* [^27^](#_ENREF_27) | ? | ? | DCV ^b, c^  FHV ^b, c^ | No [^27^](#_ENREF_27)  No [^27^](#_ENREF_27) |
| *w*Pro | *Drosophila prosaltans* [^80^](#_ENREF_80) | Male-killing likely caused by *Wolbachia* [^81^](#_ENREF_81) | ? | ? | ? | *Drosophila simulans* [^27^](#_ENREF_27) | ? | ? | DCV ^b, c^  FHV ^b, c^ | Low [^27^](#_ENREF_27)  No [^27^](#_ENREF_27) |
| *w*Sh | *Drosophila sechellia* [^82^](#_ENREF_82) | ~60% CI [^77^](#_ENREF_77) | No fecundity effect [^77^](#_ENREF_77) | ? | ? | *Drosophila simulans* [^27^](#_ENREF_27) | ? | ? | DCV ^b, c^  FHV ^b, c^ | No [^27^](#_ENREF_27)  No [^27^](#_ENREF_27) |
| *w*Stv | *Drosophila sturtevanti* [^80^](#_ENREF_80) | ? | ? | ? | ? | *Drosophila simulans* [^27^](#_ENREF_27) | ? | ? | DCV ^b, c^  FHV ^b, c^ | Moderate [^27^](#_ENREF_27)  No [^27^](#_ENREF_27) |
| *w*Tri | *Drosophila Triauraia* ^?^ | ? | ? | ? | ? | *Drosophila simulans* [^27^](#_ENREF_27) | ? | ? | DCV ^b, c^  FHV ^b, c^ | No [^27^](#_ENREF_27)  No [^27^](#_ENREF_27) |
| *w*Tro | *Drosophila tropicalis* [^83^](#_ENREF_83) | ? | ? | ? | ? | *Drosophila simulans* [^27^](#_ENREF_27) | ? | ? | DCV ^b, c^  FHV ^b, c^ | Low [^27^](#_ENREF_27)  No [^27^](#_ENREF_27) |
| *w*AlbA and *w*AlbB | *Aedes albopictus* [^84^](#_ENREF_84)^,^ [^85^](#_ENREF_85)^,^ ^[[16]](#endnote-16)^ | >99% CI [^85^](#_ENREF_85)^,^ [^86^](#_ENREF_86)^,^ [^87^](#_ENREF_87)^,^ [^88^](#_ENREF_88) (^a^ for *w*AlbA only)  >99% MT [^89^](#_ENREF_89) | Increased longevity [^86^](#_ENREF_86)^,^ [^87^](#_ENREF_87)  Increased fecundity [^86^](#_ENREF_86)^,^ [^87^](#_ENREF_87)^,^ [^90^](#_ENREF_90)  Increased hatch rate [^86^](#_ENREF_86)^,^ [^87^](#_ENREF_87)  No effect on male fitness [^91^](#_ENREF_91) | DENV ^b, c, g^    CHIKV ^b, g^ | No [^23^](#_ENREF_23)^,^ [^92^](#_ENREF_92)^,^ [^93^](#_ENREF_93)^,^ [^94^](#_ENREF_94)^,^ ^[[17]](#endnote-17)^  No [^24^](#_ENREF_24)^,^ [^95^](#_ENREF_95) | *Aedes aegypti* [^96^](#_ENREF_96)^,^ ^[[18]](#endnote-18)^ | Complete CI [^96^](#_ENREF_96)^, f^  Complete MT [^96^](#_ENREF_96)^, f^ | Reduced egg viability in quiescent state ^f^  Life-shortening ^f^  Predicted fitness costs [^96^](#_ENREF_96) | DENV ^b, c, g^ | High [^93^](#_ENREF_93) |
|  |  |  |  |  |  | *Aedes aegypti* [^97^](#_ENREF_97)^,^ ^[[19]](#endnote-19)^ | Partial CI [^97^](#_ENREF_97)  Incomplete MT [^97^](#_ENREF_97) |  |  |  |
|  |  |  |  |  |  | *Anopheles stephensi* [^98^](#_ENREF_98)^, r^ | 98% CI [^98^](#_ENREF_98)  Complete MT [^98^](#_ENREF_98) | Increased longevity when sugar-fed [^99^](#_ENREF_99)  Reduced female fecundity [^99^](#_ENREF_99)  Minor decrease in male mating competitiveness  [^99^](#_ENREF_99) | *Plasmodium falciparum* ^b, g^ | Low [^98^](#_ENREF_98) |
|  |  |  |  |  |  | *Aedes polynesiensis* [^100^](#_ENREF_100)^, r^ | Complete CI [^100^](#_ENREF_100)  Complete MT [^100^](#_ENREF_100) | Reduced survival after blood feeding [^100^](#_ENREF_100) | *Brugia pahangi* ^b^  DENV ^b, g^ | Low [^100^](#_ENREF_100)  Moderate [^101^](#_ENREF_101) |
|  |  |  |  |  |  | *Drosophila simulans* [^102^](#_ENREF_102) | >65% CI [^102^](#_ENREF_102) | ? | ? | ? |
|  |  |  |  |  |  | *Aedes albopictus* [^54^](#_ENREF_54)^, r^ and *Aedes albopictus* [^103^](#_ENREF_103)^,^ ^[[20]](#endnote-20)^ | Complete unidirectional CI, >95% bidirectional CI with *w*AlbA [^54^](#_ENREF_54) | ? | ? | ? |
| *w*RivB | 1. *Aedes riversi / Aededes polynesiensis* [^104^](#_ENREF_104)^,^ [^105^](#_ENREF_105)^,^ ^[[21]](#endnote-21)^ | Complete CI [^105^](#_ENREF_105)^,^ [^106^](#_ENREF_106) | No effect on male mating competitiveness [^105^](#_ENREF_105)^,^ [^107^](#_ENREF_107) | ? | ? | *Aedes albopictus* [^108^](#_ENREF_108) | Complete CI [^108^](#_ENREF_108)  Complete MT [^108^](#_ENREF_108) | ? | ? | ? |
| *w*Pip | *Culex pipiens* Species complex [^109^](#_ENREF_109)^,^ [^110^](#_ENREF_110) | Complete CI [^110^](#_ENREF_110)^,^ [^111^](#_ENREF_111)^,^ [^112^](#_ENREF_112)^, a^  >99% MT [^112^](#_ENREF_112) | No effect on fecundity [^112^](#_ENREF_112), or reduced fecundity and hatch rate [^113^](#_ENREF_113)  Increased longevity of blood-fed females [^113^](#_ENREF_113) | WNV ^b, g^  *Plasmodium relictum* ^b, {Zélé, 2012 #1130;Zélé, 2012 #1130}c, g^ | Low [^12^](#_ENREF_12)  Yes [^114^](#_ENREF_114)^,^ [^115^](#_ENREF_115)^,^ ^[[22]](#endnote-22)^ | *Aedes albopictus* [^116^](#_ENREF_116) | Complete CI [^103^](#_ENREF_103)^,^ [^116^](#_ENREF_116)^,^ [^117^](#_ENREF_117)  Complete MT [^116^](#_ENREF_116) | Reduced fecundity and hatch rate [^116^](#_ENREF_116)  No effect on male mating competitiveness [^117^](#_ENREF_117) | ? | ? |
|  |  |  |  |  |  | *Aedes albopictus* [^118^](#_ENREF_118)^,^ ^n^ | High CI ^[[23]](#endnote-23)^ | Minimal cost [^118^](#_ENREF_118) | ? | ? |
|  |  |  |  |  |  | *Culex pipiens* [^119^](#_ENREF_119) | >99% CI [^119^](#_ENREF_119) | ? | ? | ? |
| *w*Cer2 and *w*Cer4 | *Rhagoletis cerasi* [^120^](#_ENREF_120)^,^ ^[[24]](#endnote-24)^ | >95% CI [^120^](#_ENREF_120)^,^ [^121^](#_ENREF_121) | ? | ? | ? | *Drosophila simulans* [^122^](#_ENREF_122)^,^ ^[[25]](#endnote-25)^ | Incomplete CI [^122^](#_ENREF_122)  Incomplete MT [^122^](#_ENREF_122) | Reduced fecundity [^122^](#_ENREF_122) | ? | ? |
|  |  |  |  |  |  | *Ceratitis capitata* [^123^](#_ENREF_123)^,^ [^124^](#_ENREF_124)^,^ ^[[26]](#endnote-26)^ | Complete CI [^123^](#_ENREF_123)^,^ [^124^](#_ENREF_124)  Complete MT [^123^](#_ENREF_123)^,^ [^124^](#_ENREF_124) | ? | ? | ? |
|  |  |  |  |  |  | *Bactrocera oleae* [^125^](#_ENREF_125)^, y^ | Complete CI [^125^](#_ENREF_125)  Complete MT [^125^](#_ENREF_125) | ? | ? | ? |
| *w*Kue | *Ephestia kuehniella* [^126^](#_ENREF_126) | Partial CI [^126^](#_ENREF_126) | ? | ? | ? | *Ephestia kuehniella* [^127^](#_ENREF_127) | Partial CI [^127^](#_ENREF_127)^,^ [^128^](#_ENREF_128)  Complete MT [^127^](#_ENREF_127) | ? | ? | ? |
|  |  |  |  |  |  | *Ostrina scapulalis* [^129^](#_ENREF_129) | Complete CI [^129^](#_ENREF_129)  Incomplete MT [^129^](#_ENREF_129) | ? | ? | ? |
| *w*Sca | *Ostrinia scapulalis* [^130^](#_ENREF_130)^,^ [^131^](#_ENREF_131) | Male-killing [^132^](#_ENREF_132) [^133^](#_ENREF_133) | ? | ? | ? | *Ephestia kuehniella* [^131^](#_ENREF_131) | Partial male-killing [^129^](#_ENREF_129)^,^ [^131^](#_ENREF_131) | ? | ? | ? |
| *w*CauA and *w*CauB | *Cadra cautella* [^134^](#_ENREF_134) | Complete CI [^126^](#_ENREF_126)^,^ [^134^](#_ENREF_134)^,^ [^135^](#_ENREF_135) | ? | ? | ? | *Ephestia kuehniella* [^136^](#_ENREF_136) | Male-killing (*w*CauA) [^136^](#_ENREF_136)  Partial CI (*w*CauB) [^136^](#_ENREF_136)  Complete MT [^136^](#_ENREF_136) | ? | ? | ? |
| *w*Pre | *Trichogramma pretiosum* [^137^](#_ENREF_137) | Parthenogenesis [^137^](#_ENREF_137)^,^ [^138^](#_ENREF_138) | Reduced [^139^](#_ENREF_139) or increased [^140^](#_ENREF_140) fecundity | ? | ? | *Trichogramma dendrolimi* [^141^](#_ENREF_141) | Incomplete MT [^142^](#_ENREF_142)  Partial parthenogenesis [^141^](#_ENREF_141) | ? | ? | ? |
| *w*Den | *Trichogramma dendrolimi* ^?^ | Parthenogenesis ^?^ | ? | ? | ? | *Trichogramma evanescens* [^143^](#_ENREF_143) | Complete MT [^143^](#_ENREF_143)  No reproductive manipulation [^143^](#_ENREF_143) | ? | ? | ? |
| *w*Str | *Laodelphax striatellus* [^144^](#_ENREF_144) | Complete CI [^144^](#_ENREF_144)^,^ [^145^](#_ENREF_145) | ? | ? | ? | *Nilaparvata lugens* [^146^](#_ENREF_146) | Partial CI [^146^](#_ENREF_146)  Incomplete MT [^146^](#_ENREF_146) | ? | ? | ? |
| *w*SguBJ | *Scleroderma guani* [^147^](#_ENREF_147) | ? | ? | ? | ? | *Bemisia tabaci* [^147^](#_ENREF_147) | Complete CI [^147^](#_ENREF_147) | No fecundity effect [^147^](#_ENREF_147)  Delayed female development [^147^](#_ENREF_147) | ? | ? |
| *w*Ccep | *Corcyra cephalonica* [^148^](#_ENREF_148) | ? | ? | ? | ? | *Bemisia tabaci* [^148^](#_ENREF_148) | Complete CI [^148^](#_ENREF_148) | No fecundity effect [^148^](#_ENREF_148) | ? | ? |
| *w*Con | *Tribolium confusum* [^149^](#_ENREF_149) | Complete CI [^149^](#_ENREF_149)  Complete MT [^149^](#_ENREF_149) | Reduced female fecundity [^150^](#_ENREF_150)  Increased male fertility [^150^](#_ENREF_150) | ? | ? | *Tribolium confusum* [^151^](#_ENREF_151)^,^ [^152^](#_ENREF_152) | Complete CI [^151^](#_ENREF_151)  Complete MT [^151^](#_ENREF_151) | ? | ? | ? |
| *w*Ase | *Oniscus asellus* [^153^](#_ENREF_153) | Imperfect feminization [^154^](#_ENREF_154)  Incomplete MT [^154^](#_ENREF_154) | ? | ? | ? | *Oniscus asellus* [^154^](#_ENREF_154) | Imperfect feminization [^154^](#_ENREF_154)  Incomplete MT [^154^](#_ENREF_154) | ? | ? | ? |
| *w*VulC | *Armadillidium vulgare* [^155^](#_ENREF_155) | Feminization [^155^](#_ENREF_155)  Incomplete MT [^156^](#_ENREF_156) | Life-shortening [^157^](#_ENREF_157) | ? | ? | *Armadillium vulgare* [^157^](#_ENREF_157) | ? | ? | ? | ? |

a Level of CI declines with male age

b Level of pathogen protection determined by measurements of viral titer, bacterial load or pathogen density

c Level of pathogen protection determined by relative mortality of *Wolbachia-*infected and uninfected after exposure to pathogen

d Level of pathogen protection determined by semi-quantitative detection

e Protection against parasitoids measured by rates of egg encapsulation

f Unpublished work by JK Axford and AA Hoffmann

g Level of pathogen protection determined by estimating transmission rates (e.g. through detection in saliva or the head)

h *w*Mel does not provide protection against pathogen-induced mortality, but it greatly reduces replication

i Depends on host genetic background

j The severity of life-shortening and the strength of pathogen blockage induced by *w*MelPop increase with higher Octomom copy numbers [^158^](#_ENREF_158)

k Rates of maternal transmission depend on the male infection status

l Cell line adapted *w*MelPop (*w*MelPop-CLA)

m Reduced severity of life-shortening and strength of CI in *w*MelPop-CLA compared to *w*MelPop

n Triple infection with *w*AlbA and *w*AlbB

o Also infected naturally with *w*Stri

p Doubly infected with *w*AlbA and *w*AlbB. CI and fitness studies in *Aedes albopictus* test different combinations of infections (*w*AlbB only, *w*AlbB and *w*AlbA, or *w*AlbA only)

q *Wolbachia* in *Aedes albopictus* limits transmission ^[92](#_ENREF_92)^, but does not affect dissemination [^93^](#_ENREF_93)^,^ [^94^](#_ENREF_94) or replication of DENV [^92^](#_ENREF_92). Blagrove and others [^23^](#_ENREF_23) report that *Wolbachia* infection increases rather than limits transmission, in contrast with Mousson and others [^92^](#_ENREF_92)

r Transinfected with *w*AlbB only

s Double transinfection with *w*AlbA and *w*AlbB

t Single transinfections with both *w*AlbA and *w*AlbB

u *w*RivB originated from *Aedes riversi* but was introgressed into *Aedes polynesiensis*,

v *w*Pip protects against *Plasmodium*-induced mortality [^114^](#_ENREF_114), but increases the susceptibility of the mosquito to infection with *Plasmodium* [^115^](#_ENREF_115)

w Unpublished work by Z Xi

x Also infected with *w*Cer1, but this infection is not present in artificial transfers [^122^](#_ENREF_122). May also be infected with other *w*Cer *Wolbachia* strains [^159^](#_ENREF_159)

y Stably transinfected with *w*Cer2 only

z Both *w*Cer2 and *w*Cer4 were transferred independently into *Ceratitis capitata*, with the same effect on CI, and these two stains are bidirectionally incompatible [^123^](#_ENREF_123)

**Supplementary References**

1. Hoffmann AA, 1988. Partial cytoplasmic incompatibility between two Australian populations of *Drosophila melanogaster*. Entomologia Experimentalis et Applicata 48: 61-67.

2. Fry AJ, Palmer MR, Rand DM, 2004. Variable fitness effects of *Wolbachia* infection in *Drosophila* melanogaster. Heredity 93: 379-389.

3. Reynolds KT, Hoffmann AA, 2002. Male age, host effects and the weak expression or non-expression of cytoplasmic incompatibility in *Drosophila* strains infected by maternally transmitted *Wolbachia*. Genetical Research 80: 79-87.

4. Hoffmann AA, Hercus M, Dagher H, 1998. Population dynamics of the *Wolbachia* infection causing cytoplasmic incompatibility in *Drosophila melanogaster*. Genetics 148: 221-231.

5. Harcombe W, Hoffmann AA, 2004. *Wolbachia* effects in *Drosophila melanogaster*: in search of fitness benefits. Journal of Invertebrate Pathology 87: 45-50.

6. Montenegro H, Petherwick AS, Hurst GD, Klaczko LB, 2006. Fitness effects of *Wolbachia* and *Spiroplasma* in *Drosophila melanogaster*. Genetica 127: 207-215.

7. Serga S, Maistrenko O, Rozhok A, Mousseau T, Kozeretska I, 2014. Fecundity as one of possible factors contributing to the dominance of the *w*Mel genotype of *Wolbachia* in natural populations of *Drosophila melanogaster*. Symbiosis 63: 11-17.

8. Fry AJ, Rand DM, 2002. *Wolbachia* interactions that determine *Drosophila melanogaster* survival. Evolution 56: 1976-1981.

9. Teixeira L, Ferreira A, Ashburner M, 2008. The bacterial symbiont *Wolbachia* induces resistance to RNA viral infections in *Drosophila melanogaster*. PLoS Biology 6: e2.

10. Chrostek E, Marialva MS, Esteves SS, Weinert LA, Martinez J, Jiggins FM, Teixeira L, 2013. *Wolbachia* variants induce differential protection to viruses in *Drosophila melanogaster*: a phenotypic and phylogenomic analysis. PLoS Genetics 9: e1003896.

11. Rances E, Ye YH, Woolfit M, McGraw EA, O'Neill SL, 2012. The relative importance of innate immune priming in *Wolbachia*-mediated dengue interference. PLoS Pathogens 8: e1002548.

12. Glaser RL, Meola MA, 2010. The native *Wolbachia* endosymbionts of *Drosophila melanogaster* and *Culex quinquefasciatus* increase host resistance to West Nile virus infection. PloS One 5: e11977.

13. Panteleev DY, Goryacheva II, Andrianov BV, Reznik NL, Lazebny OE, Kulikov AM, 2007. The endosymbiotic bacterium *Wolbachia* enhances the nonspecific resistance to insect pathogens and alters behavior of *Drosophila melanogaster*. Russian Journal of Genetics 43: 1066-1069.

14. Rottschaefer SM, Lazzaro BP, 2012. No effect of *Wolbachia* on resistance to intracellular infection by pathogenic bacteria in *Drosophila melanogaster*. PLoS One 7: e40500.

15. Ye YH, Woolfit M, Rances E, O'Neill SL, McGraw EA, 2013. *Wolbachia*-associated bacterial protection in the mosquito *Aedes aegypti*. PLoS Neglected Tropical Diseases 7: e2362.

16. Martinez J, Duplouy A, Woolfit M, Vavre F, O'Neill SL, Varaldi J, 2012. Influence of the virus LbFV and of *Wolbachia* in a host-parasitoid interaction. PLoS One 7: e35081.

17. Walker T, Johnson PH, Moreira LA, Iturbe-Ormaetxe I, Frentiu FD, McMeniman CJ, Leong YS, Dong Y, Axford J, Kriesner P, Lloyd AL, Ritchie SA, O'Neill SL, Hoffmann AA, 2011. The *w*Mel *Wolbachia* strain blocks dengue and invades caged *Aedes aegypti* populations. Nature 476: 450-453.

18. Hoffmann AA, Iturbe-Ormaetxe I, Callahan AG, Phillips BL, Billington K, Axford JK, Montgomery B, Turley AP, O'Neill SL, 2014. Stability of the *w*Mel *Wolbachia* infection following invasion into *Aedes aegypti* populations. PLoS Neglected Tropical Diseases 8: e3115.

19. Ferguson NM, Hue Kien DT, Clapham H, Aguas R, Trung VT, Bich Chau TN, Popovici J, Ryan PA, O’Neill SL, McGraw EA, Long VT, Dui LT, Nguyen HL, Vinh Chau NV, Wills B, Simmons CP, 2015. Modeling the impact on virus transmission of *Wolbachia*-mediated blocking of dengue virus infection of *Aedes aegypti*. Science Translational Medicine 7: 279ra37.

20. Frentiu FD, Zakir T, Walker T, Popovici J, Pyke AT, van den Hurk A, McGraw EA, O'Neill SL, 2014. Limited dengue virus replication in field-collected *Aedes aegypti* mosquitoes infected with *Wolbachia*. PLoS Neglected Tropical Diseases 8: e2688.

21. van den Hurk AF, Hall-Mendelin S, Pyke AT, Frentiu FD, McElroy K, Day A, Higgs S, O'Neill SL, 2012. Impact of *Wolbachia* on infection with chikungunya and yellow fever viruses in the mosquito vector *Aedes aegypti*. PLoS Neglected Tropical Diseases 6: e1892.

22. Hussain M, Lu G, Torres S, Edmonds JH, Kay BH, Khromykh AA, Asgari S, 2013. Effect of *Wolbachia* on replication of West Nile virus in a mosquito cell line and adult mosquitoes. Journal of Virology 87: 851-8.

23. Blagrove MS, Arias-Goeta C, Failloux AB, Sinkins SP, 2012. *Wolbachia* strain *w*Mel induces cytoplasmic incompatibility and blocks dengue transmission in *Aedes albopictus*. Proceedings of the National Academy of Sciences of the United States of America 109: 255-260.

24. Blagrove MS, Arias-Goeta C, Di Genua C, Failloux AB, Sinkins SP, 2013. A *Wolbachia* *w*Mel transinfection in *Aedes albopictus* is not detrimental to host fitness and inhibits chikungunya virus. PLoS Neglected Tropical Diseases 7: e2152.

25. Poinsot D, Bourtzis K, Markakis G, Savakis C, Merçot H, 1998. *Wolbachia* transfer from *Drosophila melanogaster* into *D. simulans*: host effect and cytoplasmic incompatibility relationships. Genetics 150: 227-237.

26. Osborne SE, San Leong Y, O'Neill SL, Johnson KN, 2009. Variation in antiviral protection mediated by different *Wolbachia* strains in *Drosophila simulans*. PLoS Pathogens 5: e1000656.

27. Martinez J, Longdon B, Bauer S, Chan YS, Miller WJ, Bourtzis K, Teixeira L, Jiggins FM, 2014. Symbionts commonly provide broad spectrum resistance to viruses in insects: a comparative analysis of *Wolbachia* strains. PLoS Pathogens 10: e1004369.

28. Wong ZS, Hedges LM, Brownlie JC, Johnson KN, 2011. *Wolbachia*-mediated antibacterial protection and immune gene regulation in *Drosophila*. PLoS One 6: e25430.

29. Frydman HM, Li JM, Robson DN, Wieschaus E, 2006. Somatic stem cell niche tropism in *Wolbachia*. Nature 441: 509-512.

30. Min K-T, Benzer S, 1997. *Wolbachia*, normally a symbiont of *Drosophila*, can be virulent, causing degeneration and early death. Proceedings of the National Academy of Sciences 94: 10792-10796.

31. McGraw EA, Merritt DJ, Droller JN, O'Neill SL, 2001. *Wolbachia*-mediated sperm modification is dependent on the host genotype in *Drosophila*. Proceedings of the Royal Society B: Biological Sciences 268: 2565-2570.

32. Reynolds KT, Thomson LJ, Hoffmann AA, 2003. The effects of host age, host nuclear background and temperature on phenotypic effects of the virulent *Wolbachia* strain popcorn in *Drosophila melanogaster*. Genetics 164: 1027-1034.

33. McMeniman CJ, Lane AM, Fong AW, Voronin DA, Iturbe-Ormaetxe I, Yamada R, McGraw EA, O'Neill SL, 2008. Host adaptation of a *Wolbachia* strain after long-term serial passage in mosquito cell lines. Applied Environmental Microbiology 74: 6963-6969.

34. Carrington LB, Leslie J, Weeks AR, Hoffmann AA, 2009. The popcorn *Wolbachia* infection of *Drosophila melanogaster*: can selection alter *Wolbachia* longevity effects? Evolution 63: 2648-2657.

35. McGraw EA, Merritt DJ, Droller JN, O'Neill SL, 2002. *Wolbachia* density and virulence attenuation after transfer into a novel host. Proceedings of the National Academy of Sciences 99: 2918-2923.

36. Hedges LM, Brownlie JC, O'Neill SL, Johnson KN, 2008. *Wolbachia* and virus protection in insects. Science 322: 702-702.

37. McMeniman CJ, Lane RV, Cass BN, Fong AW, Sidhu M, Wang Y-F, O'Neill SL, 2009. Stable introduction of a life-shortening *Wolbachia* infection into the mosquito *Aedes aegypti*. Science 323: 141-144.

38. Yeap HL, Mee P, Walker T, Weeks AR, O'Neill SL, Johnson P, Ritchie SA, Richardson KM, Doig C, Endersby NM, Hoffmann AA, 2011. Dynamics of the "popcorn" *Wolbachia* infection in outbred *Aedes aegypti* informs prospects for mosquito vector control. Genetics 187: 583-595.

39. McMeniman CJ, O'Neill SL, 2010. A virulent *Wolbachia* infection decreases the viability of the dengue vector *Aedes aegypti* during periods of embryonic quiescence. PLoS Neglected Tropical Diseases 4: e748.

40. Turley AP, Moreira LA, O'Neill SL, McGraw EA, 2009. *Wolbachia* infection reduces blood-feeding success in the dengue fever mosquito, *Aedes aegypti*. PLoS Neglected Tropical Diseases 3: e516.

41. Moreira LA, Iturbe-Ormaetxe I, Jeffery JA, Lu G, Pyke AT, Hedges LM, Rocha BC, Hall-Mendelin S, Day A, Riegler M, Hugo LE, Johnson KN, Kay BH, McGraw EA, van den Hurk AF, Ryan PA, O'Neill SL, 2009. A Wolbachia symbiont in *Aedes aegypti* limits infection with dengue, Chikungunya, and Plasmodium. Cell 139: 1268-1278.

42. Kambris Z, Cook PE, Phuc HK, Sinkins SP, 2009. Immune activation by life-shortening *Wolbachia* and reduced filarial competence in mosquitoes. Science 326: 134-136.

43. Suh E, Mercer DR, Fu Y, Dobson SL, 2009. Pathogenicity of life-shortening *Wolbachia* in *Aedes albopictus* after transfer from *Drosophila melanogaster*. Applied Environmental Microbiology 75: 7783-7788.

44. Carrington LB, Hoffmann AA, Weeks AR, 2010. Monitoring long-term evolutionary changes following *Wolbachia* introduction into a novel host: the *Wolbachia* popcorn infection in *Drosophila simulans*. Proceedings of the Royal Society B: Biological Sciences 277: 2059-2068.

45. Holden PR, Jones P, Brookfield JF, 1993. Evidence for a *Wolbachia* symbiont in *Drosophila melanogaster*. Genetics Research 62: 23-29.

46. Veneti Z, Clark ME, Zabalou S, Karr TL, Savakis C, Bourtzis K, 2003. Cytoplasmic incompatibility and sperm cyst infection in different *Drosophila-Wolbachia* associations. Genetics 164: 545-552.

47. Yamada R, Floate KD, Riegler M, O'Neill SL, 2007. Male development time influences the strength of *Wolbachia*-induced cytoplasmic incompatibility expression in *Drosophila melanogaster*. Genetics 177: 801-808.

48. Chrostek E, Marialva MS, Yamada R, O'Neill SL, Teixeira L, 2014. High anti-viral protection without immune upregulation after interspecies *Wolbachia* transfer. PLoS One 9: e99025.

49. Hoffmann AA, Turelli M, Simmons GM, 1986. Unidirectional incompatibility between populations of *Drosophila simulans*. Evolution 40: 692-701.

50. Hoffmann AA, Turelli M, Harshman LG, 1990. Factors affecting the distribution of cytoplasmic incompatibility in *Drosophila simulans*. Genetics 126: 933-948.

51. Weeks AR, Turelli M, Harcombe WR, Reynolds KT, Hoffmann AA, 2007. From parasite to mutualist: rapid evolution of *Wolbachia* in natural populations of *Drosophila*. PLoS Biology 5: e114.

52. Turelli M, Hoffmann AA, 1995. Cytoplasmic incompatibility in *Drosophila simulans*: dynamics and parameter estimates from natural populations. Genetics 140: 1319-1338.

53. Fytrou A, Schofield PG, Kraaijeveld AR, Hubbard SF, 2006. *Wolbachia* infection suppresses both host defence and parasitoid counter-defence. Proceedings of the Royal Society B: Biological Sciences 273: 791-796.

54. Xi Z, Dean JL, Khoo C, Dobson SL, 2005. Generation of a novel *Wolbachia* infection in *Aedes albopictus* (Asian tiger mosquito) via embryonic microinjection. Insect Biochemistry and Molecular Biology 35: 903-910.

55. Fu Y, Gavotte L, Mercer DR, Dobson SL, 2010. Artificial triple *Wolbachia* infection in *Aedes albopictus* yields a new pattern of unidirectional cytoplasmic incompatibility. Applied Environmental Microbiology 76: 5887-5891.

56. Xi Z, Khoo CC, Dobson SL, 2006. Interspecific transfer of *Wolbachia* into the mosquito disease vector *Aedes albopictus*. Proceedings of the Royal Society B: Biological Sciences 273: 1317-1322.

57. Boyle L, O'Neill SL, Robertson HM, Karr TL, 1993. Interspecific and intraspecific horizontal transfer of *Wolbachia* in *Drosophila*. Science 260: 1796-1799.

58. Giordano R, O'Neill SL, Robertson HM, 1995. *Wolbachia* infections and the expression of cytoplasmic incompatibility in *Drosophila sechellia* and *D. mauritiana*. Genetics 140: 1307-1317.

59. Clancy DJ, Hoffmann AA, 1997. Behavior of *Wolbachia* endosymbionts from *Drosophila simulans* in *Drosophila serrata*, a novel host. The American Naturalist 149: 975-988.

60. Rousset F, de Stordeur E, 1994. Properties of *Drosophila simulans* strains experimentally infected by different clones of the bacterium *Wolbachia*. Heredity 72: 325-331.

61. Zabalou S, Charlat S, Nirgianaki A, Lachaise D, Mercot H, Bourtzis K, 2004. Natural *Wolbachia* infections in the *Drosophila yakuba* species complex do not induce cytoplasmic incompatibility but fully rescue the *w*Ri modification. Genetics 167: 827-834.

62. Kang L, Ma X, Cai L, Liao S, Sun L, Zhu H, Chen X, Shen D, Zhao S, Li C, 2003. Superinfection of *Laodelphax striatellus* with *Wolbachia* from *Drosophila simulans*. Heredity 90: 71-76.

63. Hoffmann AA, Clancy D, Duncan J, 1996. Naturally-occurring *Wolbachia* infection in *Drosophila simulans* that does not cause cytoplasmic incompatibility. Heredity 76: 1-8.

64. Osborne SE, Iturbe-Ormaetxe I, Brownlie JC, O'Neill SL, Johnson KN, 2012. Antiviral protection and the importance of *Wolbachia* density and tissue tropism in *Drosophila simulans*. Applied Environmental Microbiology 78: 6922-6929.

65. Yamada R, Iturbe-Ormaetxe I, Brownlie JC, O'Neill SL, 2011. Functional test of the influence of *Wolbachia* genes on cytoplasmic incompatibility expression in *Drosophila melanogaster*. Insect Molecular Biology 20: 75-85.

66. O'Neill SL, Karr TL, 1990. Bidirectional incompatibility between conspecific populations of *Drosophila simulans*. Nature 348: 170-180.

67. Mercot H, Llorente B, Jacques M, Atlan A, Montchamp-Moreau C, 1995. Variability within the *Seychelles cytoplasmic* incompatibility system in *Drosophila simulans*. Genetics 141: 1015-1023.

68. Poinsot D, Mercot H, 1997. *Wolbachia* infection in *Drosophila simulans*: does the female host bear a physiological cost? Evolution 51: 180-186.

69. Dean MD, 2006. A *Wolbachia*-associated fitness benefit depends on genetic background in *Drosophila simulans*. Proceedings of the Royal Society B: Biological Sciences 273: 1415-1420.

70. Poinsot D, Mercot H, 2001. *Wolbachia* injection from usual to naive host in *Drosophila simulans* (Diptera: Drosophilidae). European Journal of Entomology 98: 25-30.

71. Montchamp-Moreau C, Ferveur JF, Jacques M, 1991. Geographic distribution and inheritance of three cytoplasmic incompatibility types in *Drosophila simulans*. Genetics 129: 399-407.

72. Zabalou S, Apostolaki A, Pattas S, Veneti Z, Paraskevopoulos C, Livadaras I, Markakis G, Brissac T, Mercot H, Bourtzis K, 2008. Multiple rescue factors within a *Wolbachia* strain. Genetics 178: 2145-2160.

73. Dyer KA, Jaenike J, 2004. Evolutionarily stable infection by a male-killing endosymbiont in *Drosophila innubila*: molecular evidence From the host and parasite genomes. Genetics 168: 1443-1455.

74. Unckless RL, Jaenike J, 2011. Maintenence of a male-killing *Wolbachia* in *Drosophila* *innubila* by male-killing dependent and male-killing independent mechanisms. Evolution 66: 678-689.

75. Veneti Z, Zabalou S, Papafotiou G, Paraskevopoulos C, Pattas S, Livadaras I, Markakis G, Herren JK, Jaenike J, Bourtzis K, 2012. Loss of reproductive parasitism following transfer of male-killing *Wolbachia* to *Drosophila melanogaster* and *Drosophila simulans*. Heredity 109: 306-312.

76. Fast EM, Toomey ME, Panaram K, Desjardins D, Kolaczyk ED, Frydman HM, 2011. Wolbachia enhance *Drosophila* stem cell proliferation and target the germline stem cell niche. Science 334: 990-992.

77. Bourtzis K, Nirgianaki A, Markakis G, Savakis C, 1996. *Wolbachia* infection and cytoplasmic incompatibility in *Drosophila* species. Genetics 144: 1063-1073.

78. Mateos M, Castrezana SJ, Nankivell BJ, Estes AM, Markow TA, Moran NA, 2006. Heritable endosymbionts of *Drosophila*. Genetics 174: 363-376.

79. Sheeley SL, McAllister BF, 2009. Mobile male-killer: similar *Wolbachia* strains kill males of divergent *Drosophila* hosts. Heredity 102: 286-292.

80. Miller A, Riegler M, 2006. Evolutionary dynamics of *w*Au-like *Wolbachia* variants in neotropical *Drosophila* spp. Applied and Environmental Microbiology 72: 826-835.

81. Cavalcanti AL, Falcao D, Castro L, 1957. "Sex-ratio" in *Drosophila prosaltans*- a character due to interaction between nuclear genes and cytoplasmic factors. American Naturalist 91: 327-329.

82. Werren JH, Zhang W, Guo LR, 1995. Evolution and phylogeny of *Wolbachia*: reproductive parasites of arthropods. Proceedings of the Royal Society B: Biological Sciences 261: 55-63.

83. Werren JH, Windsor D, Guo L, 1995. Distribution of *Wolbachia* among neotropical arthropods. Proceedings of the Royal Society B: Biological Sciences 262: 197-204.

84. Sinkins S, Braig H, O'Neill S, 1995. *Wolbachia pipientis*: bacterial density and unidirectional cytoplasmic incompatibility between infected populations of *Aedes albopictus*. Experimental Parasitology 81: 284-291.

85. Dobson SL, Marsland EJ, Rattanadechakul W, 2001. *Wolbachia*-induced cytoplasmic incompatibility in single- and superinfected *Aedes albopictus* (Diptera: Culicidae). Journal of Medical Entomology 38: 382-387.

86. Dobson SL, Rattanadechakul W, Marsland EJ, 2004. Fitness advantage and cytoplasmic incompatibility in *Wolbachia* single- and superinfected *Aedes albopictus*. Heredity 93: 135-142.

87. Dobson SL, Marsland EJ, Rattanadechakul W, 2002. Mutualistic *Wolbachia* infection in *Aedes albopictus*: accelerating cytoplasmic drive. Genetics 160: 1087-1094.

88. Kittayapong P, Mongkalangoon P, Baimai V, O'Neill S, 2002. Host age effect and expression of cytoplasmic incompatibility in field populations of *Wolbachia*-superinfected *Aedes albopictus*. Heredity 88: 270-274.

89. Kittayapong P, Baisley KJ, Sharpe RG, Baimai V, O'Neill SL, 2002. Maternal transmission efficiency of *Wolbachia* superinfections in *Aedes albopictus* populations in Thailand. The American Journal of Tropical Medicine and Hygiene 66: 103-107.

90. Joanne S, Vythilingam I, Yugavathy N, Leong CS, Wong ML, AbuBakar S, 2015. Distribution and dynamics of *Wolbachia* infection in Malaysian *Aedes albopictus*. Acta Tropica 148: 38-45.

91. Calvitti M, Moretti R, Porretta D, Bellini R, Urbanelli S, 2009. Effects on male fitness of removing *Wolbachia* infections from the mosquito *Aedes albopictus*. Medical and Veterinary Entomology 23: 132-140.

92. Mousson L, Zouache K, Arias-Goeta C, Raquin V, Mavingui P, Failloux AB, 2012. The native *Wolbachia* symbionts limit transmission of dengue virus in *Aedes albopictus*. PLoS Neglected Tropical Diseases 6: e1989.

93. Bian G, Xu Y, Lu P, Xie Y, Xi Z, 2010. The endosymbiotic bacterium *Wolbachia* induces resistance to dengue virus in *Aedes aegypti*. PLoS Pathogens 6: e1000833.

94. Lu P, Bian G, Pan X, Xi Z, 2012. *Wolbachia* induces density-dependent inhibition to dengue virus in mosquito cells. PLoS Neglected Tropical Diseases 6: e1754.

95. Mousson L, Martin E, Zouache K, Madec Y, Mavingui P, Failloux AB, 2010. *Wolbachia* modulates Chikungunya replication in *Aedes albopictus*. Molecular Ecology 19: 1953-1964.

96. Xi Z, Khoo CC, Dobson SL, 2005. *Wolbachia* establishment and invasion in an *Aedes aegypti* laboratory population. Science 310: 326-328.

97. Ruang-Areerate T, Kittayapong P, 2006. *Wolbachia* transinfection in *Aedes aegypti*: a potential gene driver of dengue vectors. Proceedings of the National Academy of Sciences 103: 12534-12539.

98. Bian G, Joshi D, Dong Y, Lu P, Zhou G, Pan X, Xu Y, Dimopoulos G, Xi Z, 2013. Wolbachia invades *Anopheles stephensi* populations and induces refractoriness to *Plasmodium* infection. Science 340: 748-751.

99. Joshi D, McFadden MJ, Bevins D, Zhang F, Xi Z, 2014. Wolbachia strain *w*AlbB confers both fitness costs and benefit on *Anopheles stephensi*. Parasites & Vectors 7: 336.

100. Andrews ES, Crain PR, Fu Y, Howe DK, Dobson SL, 2012. Reactive oxygen species production and *Brugia pahangi* survivorship in *Aedes polynesiensis* with artificial *Wolbachia* infection types. PLoS Pathogens 8: e1003075.

101. Bian G, Zhou G, Lu P, Xi Z, 2013. Replacing a native *Wolbachia* with a novel strain results in an increase in endosymbiont load and resistance to dengue virus in a mosquito vector. PLoS Neglected Tropical Diseases 7: e2250.

102. Braig HR, Guzman H, Tesh RB, O'Neill SL, 1994. Replacement of the natural *Wolbachia* symbiont of *Drosophila simulans* with a mosquito counterpart. Nature 367: 453-455.

103. Calvitti M, 2012. *Wolbachia* strain *w*Pip yields a pattern of cytoplasmic incompatibility enhancing a *Wolbachia*-based suppression strategy against the disease vector *Aedes albopictus*. Parasites & Vectors 5: 254.

104. Wright JD, Wang B-T, 1980. Observations on Wolbachiae in mosquitoes. Journal of Invertebrate Pathology 35: 200-208.

105. Brelsfoard CL, Séchan Y, Dobson SL, 2008. Interspecific hybridization yields strategy for South Pacific filariasis vector elimination. PLoS Neglected Tropical Diseases 2: e129.

106. Dean JL, Dobson SL, 2004. Characterization of *Wolbachia* infections and interspecific crosses of *Aedes* (*Stegomyia*) *polynesiensis* and *Ae.* (*Stegomyia*) *riversi* (Diptera: Culicidae). Journal of Medical Entomology 41: 894-900.

107. Chambers EW, Hapairai L, Peel BA, Bossin H, Dobson SL, 2011. Male mating competitiveness of a *Wolbachia*-introgressed *Aedes polynesiensis* strain under semi-field conditions. PLoS Neglected Tropical Diseases 5: e1271.

108. Andrews ES, Fu Y, Calvitti M, Dobson SL, 2014. Interspecific transfer of a *Wolbachia* infection Into *Aedes albopictus* (Diptera: Culicidae) yields a novel phenotype capable of rescuing a superinfection. Journal of Medical Entomology 51: 1192-1198.

109. Hertig M, Wolbach SB, 1924. Studies on rickettsia-like micro-organisms in insects. The Journal of Medical Research 44: 329-378.

110. Yen JH, Barr AR, 1973. The etiological agent of cytoplasmic incompatibility in *Culex pipiens*. Journal of Invertebrate Pathology 22: 242-250.

111. Laven H, 1967. Eradication of *Culex pipiens fatigans* through cytoplasmic incompatibility. Nature 216: 383-384.

112. Rasgon JL, Scott TW, 2003. *Wolbachia* and cytoplasmic incompatibility in the California *Culex pipiens* mosquito species complex: parameter estimates and infection dynamics in natural populations. Genetics 165: 2029-2038.

113. Almeida Fd, Moura AS, Cardoso AF, Winter CE, Bijovsky AT, Suesdek L, 2011. Effects of *Wolbachia* on fitness of *Culex quinquefasciatus* (Diptera; Culicidae). Infection, Genetics and Evolution 11: 2138-2143.

114. Zélé F, Nicot A, Duron O, Rivero A, 2012. Infection with *Wolbachia* protects mosquitoes against *Plasmodium*‐induced mortality in a natural system. Journal of Evolutionary Biology 25: 1243-1252.

115. Zélé F, Nicot A, Berthomieu A, Weill M, Duron O, Rivero A, 2014. *Wolbachia* increases susceptibility to *Plasmodium* infection in a natural system. Proceedings of the Royal Society B: Biological Sciences 281: 20132837.

116. Calvitti M, Moretti R, Lampazzi E, Bellini R, Dobson SL, 2010. Characterization of a new *Aedes albopictus* (Diptera: Culicidae)–*Wolbachia pipientis* (Rickettsiales: Rickettsiaceae) symbiotic association generated by artificial transfer of the *w*Pip strain from *Culex pipiens* (Diptera: Culicidae). Journal of Medical Entomology 47: 179-187.

117. Moretti R, Calvitti M, 2013. Male mating performance and cytoplasmic incompatibility in a *w*Pip *Wolbachia* trans-infected line of *Aedes albopictus* (*Stegomyia albopicta*). Medical and Vetinary Entomology 27: 377-86.

118. Zhang D, Zheng X, Xi Z, Bourtzis K, Gilles JR, 2015. Combining the sterile insect technique with the incompatible insect technique: I-impact of *Wolbachia* infection on the fitness of triple- and double-infected strains of *Aedes albopictus*. PLoS One 10: e0121126.

119. Walker T, Song S, Sinkins SP, 2009. Wolbachia in the *Culex pipiens* group mosquitoes: introgression and superinfection. Journal of Heredity 100: 192-196.

120. Riegler M, Stauffer C, 2002. *Wolbachia* infections and superinfections in cytoplasmically incompatible populations of the European cherry fruit fly *Rhagoletis cerasi* (Diptera, Tephritidae). Molecular Ecology 11: 2425-2434.

121. Boller E, Bush G, 1974. Evidence for genetic variation in populations of the European cherry fruit fly, *Rhagoletis cerasi* (Diptera: Tephritidae) based on physiological parameters and hybridization experiments. Entomologia Experimentalis et Applicata 17: 279-293.

122. Riegler M, Charlat S, Stauffer C, Mercot H, 2004. *Wolbachia* transfer from *Rhagoletis cerasi* to *Drosophila simulans*: investigating the outcomes of host-symbiont coevolution. Applied and Environmental Microbiology 70: 273-279.

123. Zabalou S, Riegler M, Theodorakopoulou M, Stauffer C, Savakis C, Bourtzis K, 2004. *Wolbachia*-induced cytoplasmic incompatibility as a means for insect pest population control. Proceedings of the National Academy of Sciences 101: 15042-15045.

124. Zabalou S, Apostolaki A, Livadaras I, Franz G, Robinson AS, Savakis C, Bourtzis K, 2009. Incompatible insect technique: incompatible males from a *Ceratitis capitata* genetic sexing strain. Entomologia Experimentalis et Applicata 132: 232-240.

125. Apostolaki A, Livadaras I, Saridaki A, Chrysargyris A, Savakis C, Bourtzis K, 2011. Transinfection of the olive fruit fly *Bactrocera oleae* with *Wolbachia*: towards a symbiont-based population control strategy. Journal of Applied Entomology 135: 546-553.

126. Sasaki T, Ishikawa H, 1999. *Wolbachia* infections and cytoplasmic incompatibility in the almond moth and the Mediterranean flour moth. Zoological Science 16: 739-744.

127. Sasaki T, Ishikawa H, 2000. Transinfection of *Wolbachia* in the Mediterranean flour moth, *Ephestia kuehniella*, by embryonic microinjection. Heredity 85: 130-135.

128. Ikeda T, Ishikawa H, Sasaki T, 2003. Infection density of *Wolbachia* and level of cytoplasmic incompatibility in the Mediterranean flour moth, *Ephestia kuehniella*. Journal of Invertebrate Pathology 84: 1-5.

129. Sakamoto H, Ishikawa Y, Sasaki T, Kikuyama S, Tatsuki S, Hoshizaki S, 2005. Transinfection reveals the crucial importance of *Wolbachia* genotypes in determining the type of reproductive alteration in the host. Genetics Research 85: 205-210.

130. Kageyama D, Hoshizaki S, Ishikawa Y, 1998. Female-biased sex ratio in the Asian corn borer, *Ostrinia furnacalis*: evidence for the occurrence of feminizing bacteria in an insect. Heredity 81: 311-316.

131. Fujii Y, Kageyama D, Hoshizaki S, Ishikawa H, Sasaki T, 2001. Transfection of *Wolbachia* in Lepidoptera: the feminizer of the adzuki bean borer *Ostrinia scapulalis* causes male killing in the Mediterranean flour moth *Ephestia kuehniella*. Proceedings of the Royal Society B: Biological Sciences 268: 855-859.

132. Kageyama D, Traut W, 2004. Opposite sex-specific effects of *Wolbachia* and interference with the sex determination of its host *Ostrinia scapulalis*. Proceedings of the Royal Society B: Biological Sciences 271: 251-258.

133. Kageyama D, Nishimura G, Hoshizaki S, Ishikawa Y, 2003. Two kinds of sex ratio distorters in a moth, *Ostrinia scapulalis*. Genome 46: 974-982.

134. Kellen WR, Hoffmann DF, Kwock RA, 1981. *Wolbachia* sp. (Rickettsiales: Rickettsiaceae) a symbiont of the almond moth, *Ephestia cautella*: ultrastructure and influence on host fertility. Journal of Invertebrate Pathology 37: 273-283.

135. Brower JH, 1976. Cytoplasmic incompatibility: occurrence in a stored-product pest *Ephestia cautella*. Annals of the Entomological Society of America 69: 1011-1015.

136. Sasaki T, Kubo T, Ishikawa H, 2002. Interspecific transfer of *Wolbachia* between two lepidopteran insects expressing cytoplasmic incompatibility: a *Wolbachia* variant naturally infecting *Cadra cautella* causes male killing in *Ephestia kuehniella*. Genetics 162: 1313-1319.

137. Stouthamer R, Luck RF, Hamilton W, 1990. Antibiotics cause parthenogenetic *Trichogramma* (Hymenoptera/Trichogrammatidae) to revert to sex. Proceedings of the National Academy of Sciences 87: 2424-2427.

138. Stouthamer R, Werren JH, 1993. Microbes associated with parthenogenesis in wasps of the genus *Trichogramma*. Journal of Invertebrate Pathology 61: 6-9.

139. Stouthamer R, Luck R, 1993. Influence of microbe‐associated parthenogenesis on the fecundity of *Trichogramma deion* and *T. pretiosum*. Entomologia Experimentalis et Applicata 67: 183-192.

140. Grenier S, Gomes SM, Pintureau B, Lassablière F, Bolland P, 2002. Use of tetracycline in larval diet to study the effect of *Wolbachia* on host fecundity and clarify taxonomic status of *Trichogramma* species in cured bisexual lines. Journal of Invertebrate Pathology 80: 13-21.

141. Grenier S, Bernard P, Heddi A, Lassabliere F, Jager C, Louis C, Khatchadourian C, 1998. Successful horizontal transfer of *Wolbachia* symbionts between *Trichogramma* wasps. Proceedings of the Royal Society B: Biological Sciences 265: 1441-1445.

142. Pintureau B, Grenier S, Boleat B, Lassabliere F, Heddi A, Khatchadourian C, 2000. Dynamics of *Wolbachia* populations in transfected lines of *Trichogramma*. Journal of Invertebrate Pathology 76: 20-25.

143. Watanabe M, Kageyama D, Miura K, 2013. Transfer of a parthenogenesis-inducing *Wolbachia* endosymbiont derived from *Trichogramma dendrolimi* into *Trichogramma evanescens*. Journal of Invertebrate Pathology 112: 83-87.

144. Noda H, 1984. Cytoplasmic incompatibility in a rice planthopper. Journal of Heredity 75: 345-348.

145. Noda H, Koizumi Y, Zhang Q, Deng K, 2001. Infection density of *Wolbachia* and incompatibility level in two planthopper species, *Laodelphax striatellus* and *Sogatella furcifera*. Insect Biochemistry and Molecular Biology 31: 727-737.

146. Kawai S, Matsumoto Y, Gotoh T, Noda H, 2009. Transinfection of *Wolbachia* in planthoppers: nymphal injection of cultured *Wolbachia* and infection dynamics. Environmental Entomology 38: 1626-1633.

147. Zhong Y, Li ZX, 2014. Bidirectional cytoplasmic incompatibility induced by cross-order transfection of *Wolbachia*: implications for control of the host population. Microbial Ecology 68: 463-471.

148. Hu HY, Li ZX, 2015. A novel *Wolbachia* strain from the rice moth *Corcyra cephalonica* induces reproductive incompatibility in the whitefly *Bemisia tabaci*: sequence typing combined with phenotypic evidence. Environmental Microbiology Reports.

149. Wade MJ, Stevens L, 1985. Microorganism mediated reproductive isolation in flour beetles (genus Tribolium). Science 227: 527-528.

150. Wade MJ, Chang NW, 1995. Increased male fertility in *Tribolium confusum* beetles after infection with the intracellular parasite *Wolbachia*. Nature 373: 72-74.

151. Chang NW, Wade MJ, 1994. The transfer of *Wolbachia pipientis* and reproductive incompatibility between infected and uninfected strains of the flour beetle, *Tribolium confusum*, by microinjection. Canadian Journal of Microbiology 40: 978-981.

152. Chang NW, Wade MJ, 1996. An improved microinjection protocol for the transfer of *Wolbachia* *pipientis* between infected and uninfected strains of the flour beetle *Tribolium confusum*. Canadian Journal of Microbiology 42: 711-714.

153. Bouchon D, Rigaud T, Juchault P, 1998. Evidence for widespread *Wolbachia* infection in isopod crustaceans: molecular identification and host feminization. Proceedings of the Royal Society B: Biological Sciences 265: 1081-1090.

154. Rigaud T, Moreau J, Juchault P, 1999. *Wolbachia* infection in the terrestrial isopod *Oniscus asellus*: sex ratio distortion and effect on fecundity. Heredity 83: 469-475.

155. Rigaud T, Soutygrosset C, Raimond R, Mocquard J, Juchault P, 1991. Feminizing endocytobiosis in the terrestrial crustacean *Armadillidium vulgare* Latr (Isopoda)-recent acquisitions. Endocytobiosis and Cell Research 7: 259-273.

156. Cordaux R, Michel-Salzat A, Frelon-Raimond M, Rigaud T, Bouchon D, 2004. Evidence for a new feminizing *Wolbachia* strain in the isopod *Armadillidium vulgare*: evolutionary implications. Heredity 93: 78-84.

157. Braquart-Varnier C, Lachat M, Herbiniere J, Johnson M, Caubet Y, Bouchon D, Sicard M, 2008. *Wolbachia* mediate variation of host immunocompetence. PLoS One 3: e3286.

158. Chrostek E, Teixeira L, 2015. Mutualism breakdown by amplification of *Wolbachia* genes. PLoS Biology 13: e1002065.

159. Arthofer W, Riegler M, Schneider D, Krammer M, Miller WJ, Stauffer C, 2009. Hidden *Wolbachia* diversity in field populations of the European cherry fruit fly, *Rhagoletis cerasi* (Diptera, Tephritidae). Molecular Ecology 18: 3816-3830.

1. [↑](#endnote-ref-1)
2. [↑](#endnote-ref-2)
3. [↑](#endnote-ref-3)
4. [↑](#endnote-ref-4)
5. [↑](#endnote-ref-5)
6. [↑](#endnote-ref-6)
7. [↑](#endnote-ref-7)
8. [↑](#endnote-ref-8)
9. [↑](#endnote-ref-9)
10. [↑](#endnote-ref-10)
11. [↑](#endnote-ref-11)
12. [↑](#endnote-ref-12)
13. [↑](#endnote-ref-13)
14. [↑](#endnote-ref-14)
15. [↑](#endnote-ref-15)
16. [↑](#endnote-ref-16)
17. [↑](#endnote-ref-17)
18. [↑](#endnote-ref-18)
19. [↑](#endnote-ref-19)
20. [↑](#endnote-ref-20)
21. [↑](#endnote-ref-21)
22. [↑](#endnote-ref-22)
23. [↑](#endnote-ref-23)
24. [↑](#endnote-ref-24)
25. [↑](#endnote-ref-25)
26. [↑](#endnote-ref-26)
